# Ordinal Pattern Dependence in the Context of Long-Range Dependence

**DOI:** 10.3390/e23060670

**Published:** 2021-05-26

**Authors:** Ines Nüßgen, Alexander Schnurr

**Affiliations:** Department of Mathematics, Siegen University, Walter-Flex-Straße 3, 57072 Siegen, Germany; schnurr@mathematik.uni-siegen.de

**Keywords:** ordinal patterns, time series, long-range dependence, multivariate data analysis, limit theorems

## Abstract

Ordinal pattern dependence is a multivariate dependence measure based on the co-movement of two time series. In strong connection to ordinal time series analysis, the ordinal information is taken into account to derive robust results on the dependence between the two processes. This article deals with ordinal pattern dependence for a long-range dependent time series including mixed cases of short- and long-range dependence. We investigate the limit distributions for estimators of ordinal pattern dependence. In doing so, we point out the differences that arise for the underlying time series having different dependence structures. Depending on these assumptions, central and non-central limit theorems are proven. The limit distributions for the latter ones can be included in the class of multivariate Rosenblatt processes. Finally, a simulation study is provided to illustrate our theoretical findings.

## 1. Introduction

The origin of the concept of ordinal patterns is in the theory of dynamical systems. The idea is to consider the order of the values within a data vector instead of the full metrical information. The ordinal information is encoded as a permutation (cf. Section 3). Already in the first papers on the subject, the authors considered entropy concepts related to this ordinal structure (cf. [1]). There is an interesting relationship between these concepts and the well-known Komogorov–Sinai entropy (cf. [2,3]). Additionally, an ordinal version of the Feigenbaum diagram has been dealt with e.g., in [4]. In [5], ordinal patterns were used in order to estimate the Hurst parameter in long-range dependent time series. Furthermore, Ref. [6] have proposed a test for independence between time series (cf. also [7]). Hence, the concept made its way into the area of statistics. Instead of long patterns (or even letting the pattern length tend to infinity), rather short patterns have been considered in this new framework. Furthermore, ordinal patterns have been used in the context of ARMA processes [8] and change-point detection within one time series [9]. In [10], ordinal patterns were used for the first time in order to analyze the dependence between two time series. Limit theorems for this new concept were proven in a short-range dependent framework in [11]. Ordinal pattern dependence is a promising tool, which has already been used in financial, biological and hydrological data sets, see in this context, also [12] for an analysis of the co-movement of time series focusing on symbols. In particular, in the context of hydrology, the data sets are known to be long-range dependent. Therefore, it is important to also have limit theorems available in this framework. We close this gap in the present article.

All of the results presented in this article have been established in the Ph.D. thesis of I. Nüßgen written under the supervision of A. Schnurr.

The article is structured as follows: in the subsequent section, we provide the reader with the mathematical framework. The focus is on (multivariate) long-range dependence. In Section 3, we recall the concept of ordinal pattern dependence and prove our main results. We present a simulation study in Section 4 and close the paper by a short outlook in Section 5.

## 2. Mathematical Framework

We consider a stationary *d*-dimensional Gaussian time series ${\left(Y_{j}\right)}_{j\in Z}$ (for d∈N), with:

(1) $$\begin{array}{c} Y_{j}:={\left(Y_{j}^{\left(1\right)},\ldots ,Y_{j}^{\left(d\right)}\right)}^{t} \end{array}$$

such that $E\left(Y_{j}^{\left(p\right)}\right)=0$ and $E\left({\left(Y_{j}^{\left(p\right)}\right)}^{2}\right)=1$ for all j∈Z and p=1,…,d. Furthermore, we require the cross-correlation function to fulfil $\left|r^{\left(p,q\right)}\left(k\right)\right|<1$ for p,q=1,…,d and k≥1, where the component-wise cross-correlation functions $r^{\left(p,q\right)}\left(k\right)$ are given by $r^{\left(p,q\right)}\left(k\right)=E\left(Y_{j}^{\left(p\right)}Y_{j+k}^{\left(q\right)}\right)$ for each p,q=1,…,d and k∈Z. For each random vector $Y_{j}$, we denote the covariance matrix by $\Sigma_{d}$, since it is independent of *j* due to stationarity. Therefore, we have $\Sigma_{d}={\left(r^{\left(p,q\right)}\left(0\right)\right)}_{p,q=1,\ldots ,d}$.

We specify the dependence structure of ${\left(Y_{j}\right)}_{j\in Z}$ and turn to long-range dependence: we assume that for the cross-correlation function $r^{\left(p,q\right)}\left(k\right)$ for each p,q=1,…,d, it holds that:

(2) $$\begin{array}{c} r^{\left(p,q\right)}\left(k\right)=L_{p,q}\left(k\right)k^{d_{p}+d_{q}-1}, \end{array}$$

with $L_{p,q}\left(k\right)\to L_{p,q}$(k→∞) for finite constants $L_{p,q}\in \left[0,\infty \right)$ with $L_{p,p}\neq 0$, where the matrix $L={\left(L_{p,q}\right)}_{p,q=1,\ldots ,d}$ has full rank, is symmetric and positive definite. Furthermore, the parameters $d_{p},d_{q}\in \left(0,\frac{1}{2}\right)$ are called long-range dependence parameters. Therefore, ${\left(Y_{j}\right)}_{j\in Z}$ is multivariate long-range dependent in the sense of [13], Definition 2.1.

The processes we want to consider have a particular structure, namely for h∈N, we obtain for fixed j∈Z:

(3) $$\begin{array}{c} Y_{j,h}:={\left(Y_{j}^{\left(1\right)},\ldots ,Y_{j+h-1}^{\left(1\right)},Y_{j}^{\left(2\right)},\ldots ,Y_{j+h-1}^{\left(2\right)},\ldots ,Y_{j}^{\left(d\right)},\ldots ,Y_{j+h-1}^{\left(d\right)}\right)}^{t}\in R^{dh}. \end{array}$$

The following relation holds between the *extendend process* ${\left(Y_{j,h}\right)}_{j\in Z}$ and the primarily regarded process ${\left(Y_{j}\right)}_{j\in Z}$. For all k=1,…,dh, j∈Z we have:

(4) $$\begin{array}{c} Y_{j,h}^{\left(k\right)}=Y_{j+(k\;\mathrm{mod}\;h)-1}^{\left\lfloor \frac{k-1}{h}\right\rfloor +1}, \end{array}$$

where ⌊x⌋=max{k∈Z:k≤x}. Note that the process ${\left(Y_{j,h}\right)}_{j\in Z}$ is still a centered Gaussian process since all finite-dimensional marginals of ${\left(Y_{j}\right)}_{j\in Z}$ follow a normal distribution. Stationarity is also preserved since for all p,q=1,…,dh, p≤q and k∈Z, the cross-correlation function $r^{\left(p,q,h\right)}\left(k\right)$ of the process ${\left(Y_{j,h}\right)}_{j\in Z}$ is given by

(5) $$\begin{array}{cc} r^{\left(p,q,h\right)}\left(k\right) & =E\left(Y_{j,h}^{\left(p\right)}Y_{j+k,h}^{\left(q\right)}\right)\\ \; & =E\left(Y_{j+(p\;\mathrm{mod}\;h)-1}^{\left\lfloor \frac{p-1}{h}\right\rfloor +1}Y_{j+k+(q\;\mathrm{mod}\;h)-1}^{\left\lfloor \frac{q-1}{h}\right\rfloor +1}\right)\\ \; & =r^{\left(\left\lfloor \frac{p-1}{h}\right\rfloor +1,\left\lfloor \frac{q-1}{h}\right\rfloor +1\right)}\left(k+\left(\left(q-p\right)\;\mathrm{mod}\;h\right)\right) \end{array}$$

and the last line does not depend on *j*. The covariance matrix $\Sigma_{d,h}$ of $Y_{j,h}$ has the following structure:

Σd,hp,q=1,…,d,p≤q=r(p,q,h)(0)p,q=1,…,dh,p≤q,,Σd,hp,q=1,…,d,p>q=r(q,p,h)(0)p,q=1,…,dh,q<p.

Hence, we arrive at:

(6) $$\begin{array}{c} \Sigma_{d,h}={\left(\Sigma_{h}^{\left(p,q\right)}\right)}_{1\leq p,q\leq d}, \end{array}$$

where $\Sigma_{h}^{\left(p,q\right)}=E\left({\left(Y_{1}^{\left(p\right)},\ldots ,Y_{h}^{\left(p\right)}\right)}^{t}\left(Y_{1}^{\left(q\right)},\ldots ,Y_{h}^{\left(q\right)}\right)\right)={\left(r^{\left(p,q\right)}\left(i-k\right)\right)}_{1\leq i,k\leq h}$, p,q=1,…,d. Note that $\Sigma_{h}^{\left(p,q\right)}\in R^{h\times h}$ and $r^{\left(p,q\right)}\left(k\right)=r^{\left(q,p\right)}\left(-k\right)$, k∈Z since we are studying cross-correlation functions.

Therefore, we finally have to show that based on the assumptions on ${\left(Y_{j}\right)}_{j\in Z}$, the extended process is still long-range dependent.

Hence, we have to consider the cross-correlations again:

(7) $$\begin{array}{cc} r^{\left(p,q,h\right)}\left(k\right) & =r^{\left(\left\lfloor \frac{p-1}{h}\right\rfloor +1,\left\lfloor \frac{q-1}{h}\right\rfloor +1\right)}\left(k+\left(\left(q-p\right)\;\mathrm{mod}\;h\right)\right)\\ \; & =r^{\left(p^{*},q^{*}\right)}\left(k+m^{*}\right)\\ \; & ≃r^{\left(p^{*},q^{*}\right)}\left(k\right)\;\;\left(k\to \infty \right), \end{array}$$

since $p^{*},q^{*}\in \left\{1,\ldots ,d\right\}$ and $m^{*}\in \left\{0,\ldots ,h-1\right\}$, with $p^{*}:=\left\lfloor \frac{p-1}{h}\right\rfloor +1$, $q^{*}:=\left\lfloor \frac{q-1}{h}\right\rfloor +1$ and $m^{*}=\left(q-p\right)\;\mathrm{mod}\;h$.

Let us remark that $a_{k}≃b_{k}\Leftrightarrow \lim_{k\to \infty }\frac{a_{k}}{b_{k}}=1$.

Therefore, we are still dealing with a multivariate long-range dependent Gaussian process. We see in the proofs of the following limit theorems that the crucial parameters that determine the asymptotic distribution are the long-range dependence parameters $d_{p}$, p=1,…,d of the original process ${\left(Y_{j}\right)}_{j\in Z}$ and therefore, we omit the detailed description of the parameters $d_{p^{*}}$ herein.

It is important to remark that the extended process ${\left(Y_{j,h}\right)}_{j\in Z}$ is also long-range dependent in the sense of [14], p. 2259, since:

(8) $$\begin{array}{cc} \lim_{k\to \infty }\frac{k^{D}r^{\left(p,q,h\right)}\left(k\right)}{L(k)} & =\lim_{k\to \infty }\frac{k^{D}r^{\left(p^{*},q^{*}\right)}\left(k\right)}{L(k)}\\ \; & =\lim_{k\to \infty }\frac{k^{D}L_{p^{*},q^{*}}k^{d_{p^{*}}+d_{q^{*}}-1}}{L(k)}\\ \; & =:b_{p^{*},q^{*}}, \end{array}$$

with:

(9) $$\begin{array}{c} D:=\min_{p^{*}\in \left\{1,\ldots ,d\right\}}\left\{1-2d_{p^{*}}\right\}\in \left(0,1\right) \end{array}$$

and L(k) can be chosen as any constant $L_{p,q}$ that is not equal to zero, so for simplicity, we assume without a loss of generality $L_{1,1}\neq 0$, and therefore, $L\left(k\right)=L_{1,1}$, since the condition in [14] only requires convergence to a finite constant $b_{p^{*},q^{*}}$. Hence, we may apply the results in [14] in the subsequent results.

We define the following set, which is needed in the proofs of the theorems of this section.

(10) $$\begin{array}{c} P^{*}:=\left\{p\in \left\{1,\ldots ,d\right\}:\;d_{p}\geq d_{q},\;\mathrm{for}\;\mathrm{all}\;q\in \left\{1,\ldots ,d\right\}\right\} \end{array}$$

and denote the corresponding long-range dependence parameter to each $p\in P^{*}$ by

$$\begin{array}{c} d^{*}:=d_{p},\;p\in P^{*}. \end{array}$$

We briefly recall the concept of Hermite polynomials as they play a crucial role in determining the limit distribution of functionals of multivariate Gaussian processes.

**Definition** **1.** *(Hermite polynomial, [15], Definition 3.1)*

*The j-th Hermite polynomial $H_{j}\left(x\right)$, j=0,1,…, is defined as*

$$\begin{array}{c} H_{j}\left(x\right):={\left(-1\right)}^{j}\exp\left(\frac{x^{2}}{2}\right)\frac{d^{j}}{dx^{j}}\exp\left(-\frac{x^{2}}{2}\right). \end{array}$$

Their multivariate extension is given by the subsequent definition.

**Definition** **2.** *(Multivariate Hermite polynomial, [15], p. 122)*

*Let d∈N. We define as d-dimensional Hermite polynomial:*

$$\begin{array}{c} H_{k}\left(x\right):=H_{k_{1},\ldots ,k_{d}}\left(x\right):=H_{k_{1},\ldots ,k_{d}}\left(x_{1},\ldots ,x_{d}\right)=\prod_{j=1}^{d}H_{k_{j}}\left(x_{j}\right), \end{array}$$

*with $k=\left(k_{1},\ldots ,k_{d}\right)\in N_{0}^{d}\setminus \left\{\left(0,\ldots ,0\right)\right\}$.*

Let us remark that the case k=(0,…,0) is excluded here due to the assumption $E\left(f(X)\right)=0$.

Analogously to the univariate case, the family of multivariate Hermite polynomials

$\left\{H_{k_{1},\ldots ,k_{d}},\;k_{1},\ldots ,k_{d}\in N\right\}$ forms an orthogonal basis of $L^{2}\left(R^{d},\varphi_{I_{d}}\right)$, which is defined as

$$\begin{array}{c} L^{2}\left(R^{d},\varphi_{I_{d}}\right):=\left\{f:R^{d}\to R,\;\int_{R^{d}}f^{2}\left(x_{1},\ldots ,x_{d}\right)\varphi \left(x_{1}\right)\ldots \varphi \left(x_{d}\right)dx_{d}\ldots dx_{1}<\infty \right\}. \end{array}$$

The parameter $\varphi_{I_{d}}$ denotes the density of the *d*-dimensional standard normal distribution, which is already divided into the product of the univariate densities φ in the formula above.

We denote the Hermite coefficients by

$$\begin{array}{c} C\left(f,X,k\right):=C\left(f,I_{d},k\right):=\langle f,H_{k}\rangle =E\left(f\left(X\right)H_{k}\left(X\right)\right). \end{array}$$

The Hermite rank $m\left(f,I_{d}\right)$ of *f* with respect to the distribution $N\left(0,I_{d}\right)$ is defined as the largest integer *m*, such that:

$$\begin{array}{c} E\left(f\left(X\right)\prod_{j=1}^{d}H_{k_{j}}\left(X^{\left(j\right)}\right)\right)=0\;\mathrm{for}\;\mathrm{all}\;0<k_{1}+\ldots k_{d}<m. \end{array}$$

Having these preparatory results in mind, we derive the multivariate Hermite expansion given by

(11) $$\begin{array}{c} f\left(X\right)-Ef\left(X\right)=\sum_{k_{1}+\ldots +k_{d}\geq m\left(f,I_{d}\right)}\frac{C(f,X,k)}{k_{1}!\ldots k_{d}!}\prod_{j=1}^{d}H_{k_{j}}\left(X^{\left(j\right)}\right). \end{array}$$

We focus on the limit theorems for functionals with Hermite rank 2. First, we introduce the matrix-valued Rosenblatt process. This plays a crucial role in the asymptotics of functionals with Hermite rank 2 applied to multivariate long-range dependent Gaussian processes. We begin with the definition of a multivariate Hermitian–Gaussian random measure $\tilde{B}\left(d\lambda \right)$ with independent entries given by

(12) $$\begin{array}{c} \tilde{B}\left(d\lambda \right)={\left({\tilde{B}}^{\left(1\right)}\left(d\lambda \right),\ldots ,{\tilde{B}}^{\left(d\right)}\left(d\lambda \right)\right)}^{t}, \end{array}$$

where ${\tilde{B}}^{\left(p\right)}\left(d\lambda \right)$ is a univariate Hermitian–Gaussian random measure as defined in [16], Definition B.1.3. The multivariate Hermitian–Gaussian random measure $\tilde{B}\left(d\lambda \right)$ satisfies:

$$\begin{array}{cc} E\left(\tilde{B}\left(d\lambda \right)\right) & =0,\\ E\left(\tilde{B}\left(d\lambda \right)\tilde{B}{\left(d\lambda \right)}^{*}\right) & =I_{d}\;d\lambda \end{array}$$

and:

$$\begin{array}{cc} E\left({\tilde{B}}^{\left(p\right)}\left(d\lambda_{1}\right)\bar{{\tilde{B}}^{\left(q\right)}\left(d\lambda_{2}\right)}\right) & =0,\;\left|\lambda_{1}\right|\neq \left|\lambda_{2}\right|,\;p,q=1,\ldots ,d, \end{array}$$

where $\tilde{B}{\left(d\lambda \right)}^{*}=\left(\bar{B^{\left(1\right)}\left(d\lambda \right)},\ldots ,\bar{B^{\left(d\right)}\left(d\lambda \right)}\right)$ denotes the Hermitian transpose of $\tilde{B}\left(d\lambda \right)$. Thus, following [14], Theorem 6, we can state the spectral representation of the matrix-valued Rosenblatt process $Z_{2,H}\left(t\right)$, t∈[0,1] as

$$\begin{array}{c} Z_{2,H}\left(t\right)={\left(Z_{2,H}^{\left(p,q\right)}\left(t\right)\right)}_{p,q=1,\ldots ,d} \end{array}$$

where each entry of the matrix is given by

$$\begin{array}{c} Z_{2,H}^{\left(p,q\right)}\left(t\right)=\int_{R^{2}}^{\prime \prime }\frac{\exp\left(it\left(\lambda_{1}+\lambda_{2}\right)\right)-1}{i\left(\lambda_{1}+\lambda_{2}\right)}{\tilde{B}}^{\left(p\right)}\left(d\lambda_{1}\right){\tilde{B}}^{\left(q\right)}\left(d\lambda_{2}\right). \end{array}$$

The double prime in $\int_{R^{2}}^{\prime \prime }$ excludes the diagonals $\left|\lambda_{i}\right|=\left|\lambda_{j}\right|$, i≠j in the integration. For details on multiple Wiener-Itô integrals, as can be seen in [17].

The following results were taken from [18], Section 3.2. The corresponding proofs were outsourced to the Appendix A.

**Theorem** **1.** *Let ${\left(Y_{j}\right)}_{j\in Z}$ be a stationary Gaussian process as defined in (1) that fulfils (2) for $d_{p}\in \left(\frac{1}{4},\frac{1}{2}\right)$, p=1…,d. For h∈N we fix:*

$$\begin{array}{c} Y_{j,h}:={\left(Y_{j}^{\left(1\right)},\ldots ,Y_{j+h-1}^{\left(1\right)},\ldots ,Y_{j}^{\left(d\right)},\ldots ,Y_{j+h-1}^{\left(d\right)}\right)}^{t}\in R^{dh} \end{array}$$

*with $Y_{j,h}∼N\left(0,\Sigma_{d,h}\right)$ and $\Sigma_{d,h}$ as described in (6). Let $f:R^{dh}\to R$ be a function with Hermite rank 2 such that the set of discontinuity points $D_{f}$ is a Null set with respect to the dh-dimensional Lebesgue measure. Furthermore, we assume f fulfills $E\left(f^{2}\left(Y_{j,h}\right)\right)<\infty$. Then:*

$$\begin{array}{cc} n^{-2d^{*}}{\left(C_{2}\right)}^{-\frac{1}{2}} & \sum_{j=1}^{n}\left(f\left(Y_{j}^{\left(1\right)},\ldots ,Y_{j+h-1}^{\left(d\right)}\right)-E\left(f\left(Y_{j}^{\left(1\right)},\ldots ,Y_{j+h-1}^{\left(d\right)}\right)\right)\right) \end{array}$$

(13) $$\begin{array}{c} \overset{D}{\to }\sum_{p,q\in P^{*}}{\tilde{\alpha }}^{\left(p,q\right)}Z_{2,d^{*}+1/2}^{\left(p,q\right)}\left(1\right), \end{array}$$

*where:*

$$\begin{array}{c} Z_{2,d^{*}+1/2}^{\left(p,q\right)}\left(1\right)=K_{p,q}\left(d^{*}\right)\int_{R^{2}}^{\prime \prime }\frac{\exp\left(i\left(\lambda_{1}+\lambda_{2}\right)\right)-1}{i\left(\lambda_{1}+\lambda_{2}\right)}{\left|\lambda_{1}\lambda_{2}\right|}^{-d^{*}}{\tilde{B}}_{L}^{\left(p\right)}\left(d\lambda_{1}\right){\tilde{B}}_{L}^{\left(q\right)}\left(d\lambda_{2}\right). \end{array}$$

The matrix $K\left(d^{*}\right)$ is a normalizing constant, as can be seen in [18], Corollary 3.6. Moreover, ${\tilde{B}}_{L}\left(d\lambda \right)$ is a multivariate Hermitian–Gaussian random measure with $E\left(B_{L}\left(d\lambda \right)B_{L}{\left(d\lambda \right)}^{*}\right)=L\;d\lambda$ and L as defined in (2). Furthermore, $C_{2}:=\frac{1}{2d^{*}\left(4d^{*}-1\right)}$ is a normalizing constant and:

$$\begin{array}{c} {\tilde{\alpha }}^{\left(p,q\right)}:=\sum_{i,k=1}^{h}\alpha_{i,k}^{\left(p,q\right)} \end{array}$$

where $\alpha_{i,k}^{\left(p,q\right)}=\alpha_{i+(p-1)h,k+(q-1)h}$ for each $p,q\in P^{*}$ and i,k=1,…,h and:

$$\begin{array}{c} {\left(\alpha_{i,k}\right)}_{1\leq i,k\leq dh}=\Sigma_{d,h}^{-1}C\Sigma_{d,h}^{-1} \end{array}$$

*where C denotes the matrix of second order Hermite coefficients, given by*

$$\begin{array}{c} C={\left(c_{i,k}\right)}_{1\leq i,k\leq dh}=E\left(Y_{1,h}\left(f\left(Y_{1,h}\right)-E\left(f\left(Y_{1,h}\right)\right)\right)Y_{1,h}^{t}\right). \end{array}$$

It is possible to soften the assumptions in Theorem 1 to allow for mixed cases of short- and long-range dependence.

**Corollary** **1.** *Instead of demanding in the assumptions of Theorem 1 that (2) holds for ${\left(Y_{j}\right)}_{j\in Z}$ with the addition that for all p=1,…,d we have $d_{p}\in \left(\frac{1}{4},\frac{1}{2}\right)$, we may use the following condition.*

*We assume that:*

$$\begin{array}{c} r^{\left(p,q\right)}\left(k\right)=k^{d_{p}+d_{q}-1}L_{p,q}\left(k\right)\;\left(k\to \infty \right) \end{array}$$

with $L_{p,q}\left(k\right)$ as given in (2), but we do no longer assume $d_{p}\in \left(\frac{1}{4},\frac{1}{2}\right)$ for all p=1,…,d but soften the assumption to $d^{*}\in \left(\frac{1}{4},\frac{1}{2}\right)$ and for $d_{p}\neq d^{*}$, p=1,…,d we allow for $d_{p}\in \left(-\infty ,0\right)\cup \left(0,\frac{1}{4}\right]$. Then, the statement of Theorem 1 remains valid.

However, with a mild technical assumption on the covariances of the one-dimensional marginal Gaussian processes that is often fulfilled in applications, there is another way of normalizing the partial sum on the right-hand side in Theorem 1, this time explicitly for the case $\#P^{*}=2$ and h∈N, such that the limit can be expressed in terms of two standard Rosenblatt random variables. This yields the possibility of further studying the dependence structure between these two random variables. In the following theorem, we assume $\#P^{*}=d=2$ for the reader’s convenience.

**Theorem** **2.** Under the same assumptions as in Theorem 1 with $\#P^{*}=d=2$ and $d^{*}\in \left(\frac{1}{4},\frac{1}{2}\right)$ and the additional condition that $r^{\left(1,1\right)}\left(l\right)=r^{\left(2,2\right)}\left(l\right)$, for l=0,…,h−1, and $L_{1,1}+L_{2,2}\neq L_{1,2}+L_{2,1}$, it holds that:

$$\begin{array}{cc} n^{-2d^{*}}{\left(C_{2}\right)}^{-\frac{1}{2}} & \sum_{j=1}^{n}\left(f\left(Y_{j}^{\left(1\right)},\ldots ,Y_{j+h-1}^{\left(d\right)}\right)-Ef\left(Y_{j}^{\left(1\right)},\ldots ,Y_{j+h-1}^{\left(d\right)}\right)\right)\\ \; & \overset{D}{\to }\left({\tilde{\alpha }}^{\left(1,1\right)}-{\tilde{\alpha }}^{\left(1,2\right)}\right)\frac{L_{2,2}-L_{2,1}-L_{1,2}+L_{1,1}}{2}Z_{2,d^{*}+1/2}^{*}\left(1\right)\\ \; & \;\;+\left({\tilde{\alpha }}^{\left(1,1\right)}+{\tilde{\alpha }}^{\left(1,2\right)}\right)\frac{L_{2,2}+L_{2,1}+L_{1,2}+L_{1,1}}{2}Z_{2,d^{*}+1/2}^{**}\left(1\right) \end{array}$$

with $C_{2}:=\frac{1}{2d^{*}\left(4d^{*}-1\right)}$ being the same normalizing factor as in Theorem 1, ${\left(\alpha_{i,k}\right)}_{1\leq i,k\leq dh}=\Sigma_{d,h}^{-1}C\Sigma_{d,h}^{-1}$ and $C={\left(c_{i,k}\right)}_{1\leq i,k\leq dh}=E\left(Y_{1,h}\left(f\left(Y_{1,h}\right)-Ef\left(Y_{1,h}\right)\right)Y_{1,h}^{t}\right)$. Note that $Z_{2,d^{*}+1/2}^{*}\left(1\right)$ and $Z_{2,d^{*}+1/2}^{**}\left(1\right)$ are both standard Rosenblatt random variables whose covariance is given by

(14) $$\begin{array}{c} Cov\left(Z_{2,d^{*}+1/2}^{*}\left(1\right),Z_{2,d^{*}+1/2}^{**}\left(1\right)\right)=\frac{{\left(L_{2,2}-L_{1,1}\right)}^{2}}{{\left(L_{1,1}+L_{2,2}\right)}^{2}-{\left(L_{1,2}+L_{2,1}\right)}^{2}}. \end{array}$$

## 3. Ordinal Pattern Dependence

Ordinal pattern dependence is a multivariate dependence measure that compares the co-movement of two time series based on the ordinal information. First introduced in [10] to analyze financial time series, a mathematical framework including structural breaks and limit theorems for functionals of absolutely regular processes has been built in [11]. In [19], the authors have used the so-called symbolic correlation integral in order to detect the dependence between the components of a multivariate time series. Their considerations focusing on testing independence between two time series are also based on ordinal patterns. They provide limit theorems in the i.i.d.-case and otherwise use bootstrap methods. In contrast, in the mathematical model in the present article, we focus on asymptotic distributions of an estimator of ordinal pattern dependence having a bivariate Gaussian time series in the background but allowing for several dependence structures to arise. As it will turn out in the following, this yields central but also non-central limit theorems.

We start with the definition of an ordinal pattern and the basic mathematical framework that we need to build up the ordinal model.

Let $S_{h}$ denote the set of permutations in {0,…,h}, $h\in N_{0}$ that we express as (h+1)-dimensional tuples, assuring that each tuple contains each of the numbers above exactly once. In mathematical terms, this yields:

$$\begin{array}{c} S_{h}=\left\{\pi \in N_{0}^{h+1}:\;0\leq \pi_{i}\leq h,\;\mathrm{and}\;\pi_{i}\neq \pi_{k},\;\mathrm{whenever}\;i\neq k,\;i,k=0,\ldots ,h\right\}, \end{array}$$

as can be seen in [11], Section 2.1.

The number of permutations in $S_{h}$ is given by $\#S_{h}=\left(h+1\right)!$. In order to get a better intuitive understanding of the concept of ordinal patterns, we have a closer look at the following example, before turning to the formal definition.

**Figure 1:**
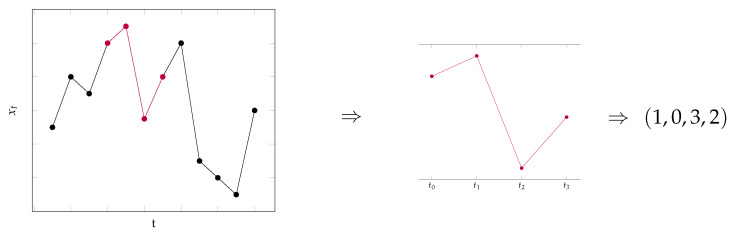
Example of the extraction of an ordinal pattern of a given data set.

**Example** **1.** *Figure 1 provides an illustrative understanding of the extraction of an ordinal pattern from a data set. The data points of interest are colored in red and we consider a pattern of length h=3, which means that we have to take n=4 data points into consideration. We fix the points in time $t_{0}$, $t_{1}$, $t_{2}$ and $t_{3}$ and extract the data points from the time series. Then, we search for the point in time which exhibits the largest value in the resulting data and write down the corresponding time index. In this example, it was given by $t=t_{1}$. We order the data points by writing the time position of the largest value as the first entry, the time position of the second largest as the second entry, etc. Hence, the absolute values are ordered from largest to smallest and the ordinal pattern $\left(1,0,3,2\right)\in S_{3}$ is obtained for the considered data points.*

Formally, the aforementioned procedure can be defined as follows, as can be seen in [11], Section 2.1.

**Definition** **3.** *As the ordinal pattern of a vector $x=\left(x_{0},\ldots ,x_{h}\right)\in R^{h+1}$, we define the unique permutation $\pi =\left(\pi_{0},\ldots ,\pi_{h}\right)\in S_{h}$:*

$$\begin{array}{c} \Pi \left(x\right)=\Pi \left(x_{0},\ldots ,x_{h}\right)=\left(\pi_{0},\ldots ,\pi_{h}\right), \end{array}$$

*such that:*

$$\begin{array}{c} x_{\pi_{0}}\geq \ldots \geq x_{\pi_{h}}, \end{array}$$

*with $\pi_{i-1}<\pi_{i}$ if $x_{\pi_{i-1}}=x_{\pi_{i}}$, i=1,…,h.*

The last condition assures the uniqueness of π if there are ties in the data sets. In particular, this condition is necessary if real-world data are to be considered.

**Figure 2:**
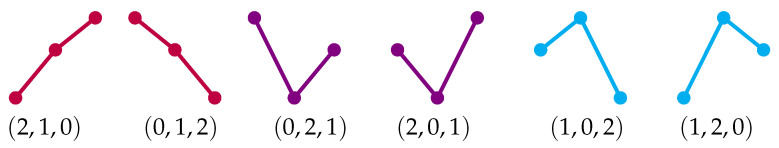
Ordinal patterns for h=2.

In Figure 2, all ordinal patterns of length h=2 are shown. As already mentioned in the introduction, from the practical point of view, a highly desirable property of ordinal patterns is that they are not affected by monotone transformations, as can be seen in [5], p. 1783.

Mathematically, this means that if f:R→R is strictly monotone, then:

(15) $$\begin{array}{c} \Pi \left(x_{0},\ldots ,x_{h}\right)=\Pi \left(f\left(x_{0}\right),\ldots ,f\left(x_{h}\right)\right). \end{array}$$

In particular, this includes linear transformations f(x)=ax+b, with $a\in R^{+}$ and b∈R.

Following [11], Section 1, the minimal requirement of the data sets we use for ordinal analysis in the time series context, i.e., for ordinal pattern probabilities as well as for ordinal pattern dependence later on, is *ordinal pattern stationarity (of order h)*. This property implies that the probability of observing a certain ordinal pattern of length *h* remains the same when shifting the moving window of length *h* through the entire time series and is not depending on the specific points in time. In the course of this work, the time series, in which the ordinal patterns occur, always have either stationary increments or are even stationary themselves. Note that both properties imply ordinal pattern stationarity. The reason why requiring stationary increments is a sufficient condition is given in the following explanation.

One fundamental property of ordinal patterns is that they are uniquely determined by the increments of the considered time series. As one can imagine in Example 1, the knowledge of the increments between the data points is sufficient to obtain the corresponding ordinal pattern. In mathematical terms, we can define another mapping $\tilde{\Pi }$, which assigns the corresponding ordinal pattern to each vector of increments, as can be seen in [5], p. 1783.

**Definition** **4.** *We define for $y=\left(y_{1},\ldots ,y_{h}\right)\in R^{h}$ the mapping $\tilde{\Pi }:R^{h}\to S_{h}$:*

$$\begin{array}{c} \tilde{\Pi }\left(y_{1},\ldots ,y_{h}\right):=\Pi \left(0,y_{1},y_{1}+y_{2},\ldots ,y_{1}+\ldots +y_{h}\right), \end{array}$$

*such that for $y_{i}=x_{i}-x_{i-1}$, i=1,…,h, we obtain:*

$$\begin{array}{cc} \tilde{\Pi }\left(y_{1},\ldots ,y_{h}\right) & =\Pi \left(0,y_{1},y_{1}+y_{2},\ldots ,y_{1}+\ldots +y_{h}\right)\\ \; & =\Pi \left(0,x_{1}-x_{0},x_{2}-x_{0},\ldots ,x_{h}-x_{0}\right)\\ \; & =\Pi \left(x_{0},x_{1},x_{2},\ldots ,x_{h}\right). \end{array}$$

We define the two mappings, following [5], p. 1784:

$$\begin{array}{cc} \; & S:S_{h}\to S_{h},\left(\pi_{0},\ldots ,\pi_{h}\right)\to \left(\pi_{h},\ldots ,\pi_{0}\right),\\ \; & T:S_{h}\to S_{h},\left(\pi_{0},\ldots ,\pi_{h}\right)\to \left(h-\pi_{0},\ldots ,h-\pi_{h}\right). \end{array}$$

**Figure 3:**
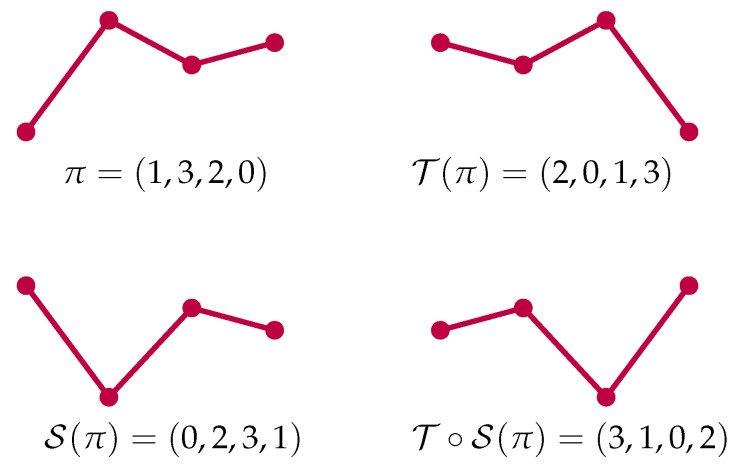
Space and time reversion of the pattern π=(1,3,2,0).

An illustrative understanding of these mappings is given as follows. The mapping S(π), which is the spatial reversion of the pattern π, is the reflection of π on a horizontal line, while T(π), the time reversal of π, is its reflection on a vertical line, as one can observe in Figure 3.

Based on the spatial reversion, we define a possibility to divide $S_{h}$ into two disjoint sets.

**Definition** **5.** We define $S_{h}^{*}$ as a subset of $S_{h}$ with the property that for each $\pi \in S_{h}$, either π or S(π) are contained in the set, but not both of them.

Note that this definition does not yield the uniqueness of $S_{h}^{*}$.

**Example** **2.** We consider the case h=2 again and we want to divide $S_{2}$ into a possible choice of $S_{2}^{*}$ and the corresponding spatial reversal. We choose $S_{2}^{*}=\left\{\left(2,1,0\right),\left(2,0,1\right),\left(1,2,0\right)\right\}$, and therefore, $S_{2}\setminus S_{2}^{*}=\left\{\left(0,1,2\right),\left(1,0,2\right),\left(0,2,1\right)\right\}$. Remark that $S_{2}^{*}=\left\{\left(0,1,2\right),\left(2,0,1\right),\left(1,2,0\right)\right\}$ is also a possible choice. The only condition that has to be satisfied is that if one permutation is chosen for $S_{2}^{*}$, then its spatial reverse must not be an element of this set.

We stick to the formal definition of ordinal pattern dependence, as it is proposed in [11], Section 2.1. The considered moving window consists of h+1 data points, and hence, *h* increments. We define:

(16) $$\begin{array}{cc} p: & =p_{X^{\left(1\right)},X^{\left(2\right)}}:=P\left(\Pi \left(X_{0}^{\left(1\right)},\ldots ,X_{h}^{\left(1\right)}\right)=\Pi \left(X_{0}^{\left(2\right)},\ldots ,X_{h}^{\left(2\right)}\right)\right) \end{array}$$

and:

$$\begin{array}{cc} q: & =q_{X^{\left(1\right)},X^{\left(2\right)}}:=\sum_{\pi \in S_{h}}P\left(\Pi \left(X_{0}^{\left(1\right)},\ldots ,X_{h}^{\left(1\right)}\right)=\pi \right)P\left(\Pi \left(X_{0}^{\left(2\right)},\ldots ,X_{h}^{\left(2\right)}\right)=\pi \right). \end{array}$$

Then, we define ordinal pattern dependence OPD as

(17) $$\begin{array}{c} OPD:=OPD_{X^{\left(1\right)},X^{\left(2\right)}}:=\frac{p-q}{1-q}. \end{array}$$

The parameter *q* represents the hypothetical case of independence between the two time series. In this case, *p* and *q* would obtain equal values and therefore, OPD would equal zero. Regarding the other extreme, the case in which both processes coincide or one is a strictly monotone increasing transform of the other one, we obtain the value 1. However, in the following, we assume p∈(0,1) and q∈(0,1).

Note that the definition of ordinal pattern dependence in (17) only measures positive dependence. This is no restriction in practice, because negative dependence can be investigated in an analogous way, by considering $OPD_{X^{\left(1\right)},-X^{\left(2\right)}}$. If one is interested in both types of dependence simultaneously, in [11], the authors propose to use ${\left(OPD_{X^{\left(1\right)},X^{\left(2\right)}}\right)}_{+}-{\left(OPD_{X^{\left(1\right)},-X^{\left(2\right)}}\right)}_{+}$. To keep the notation simple, we focus on OPD as it is defined in (17).

We compare whether the ordinal patterns in ${\left(X_{j}^{\left(1\right)}\right)}_{j\in Z}$ coincide with the ones in ${\left(X_{j}^{\left(2\right)}\right)}_{j\in Z}$. Recall that it is an essential property of ordinal patterns that they are uniquely determined by the increment process. Therefore, we have to consider the increment processes ${\left(Y_{j}\right)}_{j\in Z}={\left(\left(Y_{j}^{\left(1\right)},Y_{j}^{\left(2\right)}\right)\right)}_{j\in Z}$ as defined in (1) for d=2, where $Y_{j}^{\left(p\right)}=X_{j}^{\left(p\right)}-X_{j-1}^{\left(p\right)}$, p=1,2. Hence, we can also express *p* and *q* (and consequently OPD) as a probability that only depends on the increments of the considered vectors of the time series. Recall the definition of ${\left(Y_{j,h}\right)}_{j\in Z}$ for d=2, given by

$$\begin{array}{c} Y_{j,h}={\left(Y_{j}^{\left(1\right)},\ldots ,Y_{j+h-1}^{\left(1\right)},Y_{j}^{\left(2\right)},\ldots ,Y_{j+h-1}^{\left(2\right)}\right)}^{t}, \end{array}$$

such that $Y_{j,h}∼N\left(0,\Sigma_{2,h}\right)$ with $\Sigma_{2,h}$ as given in (6).

In the course of this article, we focus on the estimation of *p*. For a detailed investigation of the limit theorems for estimators of OPD, we refer to [18]. We define the estimator of *p*, the probability of coincident patterns in both time series in a moving window of fixed length, by

$$\begin{array}{cc} {\hat{p}}_{n} & =\frac{1}{n-h}\sum_{j=0}^{n-h-1}1_{\left\{\Pi \left(X_{j}^{\left(1\right)},\ldots ,X_{j+h}^{\left(1\right)}\right)=\Pi \left(X_{j}^{\left(2\right)},\ldots ,X_{j+h}^{\left(2\right)}\right)\right\}}\\ \; & =\frac{1}{n-h}\sum_{j=1}^{n-h}1_{\left\{\tilde{\Pi }\left(Y_{j}^{\left(1\right)},\ldots ,Y_{j+h-1}^{\left(1\right)}\right)=\tilde{\Pi }\left(Y_{j}^{\left(2\right)},\ldots ,Y_{j+h-1}^{\left(2\right)}\right)\right\}}, \end{array}$$

where:

$$\begin{array}{cc} \tilde{\Pi }\left(Y_{1},\ldots ,Y_{h}\right): & =\Pi \left(0,Y_{1},Y_{1}+Y_{2},\ldots ,Y_{1}+\ldots +Y_{h}\right)\\ \; & =\Pi \left(0,X_{1}-X_{0},\ldots ,X_{h}-X_{0}\right)\\ \; & =\Pi \left(X_{0},X_{1},\ldots ,X_{h}\right). \end{array}$$

**Figure 4:**
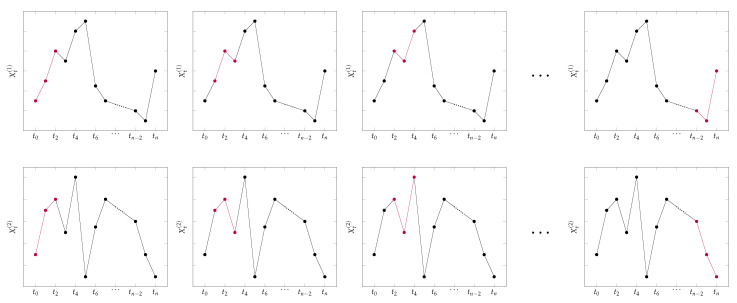
Illustration of estimation of ordinal pattern dependence.

Figure 4 illustrates the way ordinal pattern dependence is estimated by ${\hat{p}}_{n}$. The patterns of interest that are compared in each moving window are colored in red.

Having emphasized the crucial importance of the increments, we define the following conditions on the increment process ${\left(Y_{j}\right)}_{j\in Z}$: let ${\left(Y_{j}\right)}_{j\in Z}$ be a bivariate, stationary Gaussian process with $Y_{j}^{\left(p\right)}∼N\left(0,1\right)$, p=1,2:

**(L)**  We assume that ${\left(Y_{j}\right)}_{j\in Z}$ fulfills (2) with $d^{*}$ in $\left(\frac{1}{4},\frac{1}{2}\right)$. We allow for $\min\left\{d_{1},d_{2}\right\}$ to be in the range $\left(-\infty ,0\right)\cup \left(0,\frac{1}{4}\right]$.
**(S)**  We assume $d_{1},d_{2}\in \left(-\infty ,0\right)\cup \left(0,\frac{1}{4}\right)$ such that the cross-correlation function of ${\left(Y_{j}\right)}_{j\in Z}$ fulfills for p,q=1,2:
  $$\begin{array}{c} r^{\left(p,q\right)}\left(k\right)=k^{d_{p}+d_{q}-1}L_{p,q}\left(k\right)\;\left(k\to \infty \right) \end{array}$$
  with $L_{p,q}\left(k\right)\to L_{p,q}$ and $L_{p,q}\in R$ holds.

Furthermore, in both cases, it holds that $\left|r^{\left(p,q\right)}\left(k\right)\right|<1$ for p,q=1,2 and k≥1 to exclude ties.

We begin with the investigation of the asymptotics of ${\hat{p}}_{n}$. First, we calculate the Hermite rank of ${\hat{p}}_{n}$, since the Hermite rank determines for which ranges of $d^{*}$ the estimator ${\hat{p}}_{n}$ is still long-range dependent. Depending on this range, different limit theorems may hold.

**Lemma** **1.** *The Hermite rank of $f\left(Y_{j,h}\right)=1_{\left\{\tilde{\Pi }\left(Y_{j+1}^{\left(1\right)},\ldots ,Y_{j+h}^{\left(1\right)}\right)=\tilde{\Pi }\left(Y_{j+1}^{\left(2\right)},\ldots ,Y_{j+h}^{\left(2\right)}\right)\right\}}$ with respect to $\Sigma_{2,h}$ is equal to 2.*

**Proof.**  Following [20], Lemma 5.4 it is sufficient to show the following two properties:

(i) $m(f,\Sigma_{2,h})\geq 2$,
(ii) $m(f,I_{2,h})\leq 2$.

Note that the conclusion is not trivial, because $m\left(f,\Sigma_{2,h}\right)\neq m\left(f,I_{2,h}\right)$ in general, as can be seen in [15], Lemma 3.7. Lemma 5.4 in [20] can be applied due to the following reasoning. Ordinal patterns are not affected by scaling, therefore, the technical condition that $\Sigma_{2,h}^{-1}-I_{2,h}$ is positive semidefinite is fulfilled in our case. We can scale the standard deviation of the random vector $Y_{j,h}$ by any positive real number σ>0 since for all j∈Z we have:

$$\begin{array}{c} \left\{\tilde{\Pi }\left(Y_{j}^{\left(1\right)},\ldots ,Y_{j+h-1}^{\left(1\right)}\right)=\tilde{\Pi }\left(Y_{j}^{\left(2\right)},\ldots ,Y_{j+h-1}^{\left(2\right)}\right)\right\}\\ =\left\{\tilde{\Pi }\left(\sigma Y_{j}^{\left(1\right)},\ldots ,\sigma Y_{j+h-1}^{\left(1\right)}\right)=\tilde{\Pi }\left(\sigma Y_{j}^{\left(2\right)},\ldots ,\sigma Y_{j+h-1}^{\left(2\right)}\right)\right\}. \end{array}$$

To show property (i), we need to consider a multivariate random vector:

$$\begin{array}{c} Y_{1,h}:={\left(Y_{1}^{\left(1\right)},\ldots ,Y_{h}^{\left(1\right)},Y_{1}^{\left(2\right)},\ldots ,Y_{h}^{\left(2\right)}\right)}^{t} \end{array}$$

with covariance matrix $\Sigma_{2,h}$. We fix i=1,…,2h. We divide the set $S_{h}$ into disjoint sets, namely into $S_{h}^{*}$, as defined in Definition 5 and the complimentary set $S_{h}\setminus S_{h}^{*}$. Note that:

$$\begin{array}{c} -Y_{j,h}\overset{D}{=}Y_{j,h} \end{array}$$

holds. This implies:

$$\begin{array}{c} E\left(Y_{j,h}^{\left(i\right)}1_{\left\{\tilde{\Pi }\left(Y_{1}^{\left(1\right)},\ldots ,Y_{h}^{\left(1\right)}\right)=\tilde{\Pi }\left(Y_{1}^{\left(2\right)},\ldots ,Y_{h}^{\left(2\right)}\right)=\pi \right\}}\right)=-E\left(Y_{j,h}^{\left(i\right)}1_{\left\{\tilde{\Pi }\left(Y_{1}^{\left(1\right)},\ldots ,Y_{h}^{\left(1\right)}\right)=\tilde{\Pi }\left(Y_{1}^{\left(2\right)},\ldots ,Y_{h}^{\left(2\right)}\right)=S\left(\pi \right)\right\}}\right) \end{array}$$

for $\pi \in S_{h}$. Hence, we arrive at:

$$\begin{array}{cc} E\left(Y_{j,h}^{\left(i\right)}f\left(Y_{j,h}\right)\right) & =E\left(Y_{j,h}^{\left(i\right)}1_{\left\{\tilde{\Pi }\left(Y_{1}^{\left(1\right)},\ldots ,Y_{h}^{\left(1\right)}\right)=\tilde{\Pi }\left(Y_{1}^{\left(2\right)},\ldots ,Y_{h}^{\left(2\right)}\right)\right\}}\right)\\ \; & =\sum_{\pi \in S_{h}}E\left(Y_{j,h}^{\left(i\right)}1_{\left\{\tilde{\Pi }\left(Y_{1}^{\left(1\right)},\ldots ,Y_{h}^{\left(1\right)}\right)=\tilde{\Pi }\left(Y_{1}^{\left(2\right)},\ldots ,Y_{h}^{\left(2\right)}\right)=\pi \right\}}\right)\\ \; & =\sum_{\pi \in S_{h}^{*}}E\left(Y_{j,h}^{\left(i\right)}1_{\left\{\tilde{\Pi }\left(Y_{1}^{\left(1\right)},\ldots ,Y_{h}^{\left(1\right)}\right)=\tilde{\Pi }\left(Y_{1}^{\left(2\right)},\ldots ,Y_{h}^{\left(2\right)}\right)=\pi \right\}}\right)\\ & \;\;-\sum_{\pi \in S_{h}\setminus S_{h}^{*}}E\left(Y_{j,h}^{\left(i\right)}1_{\left\{\tilde{\Pi }\left(Y_{1}^{\left(1\right)},\ldots ,Y_{h}^{\left(1\right)}\right)=\tilde{\Pi }\left(Y_{1}^{\left(2\right)},\ldots ,Y_{h}^{\left(2\right)}\right)=S\left(\pi \right)\right\}}\right)\\ \; & =0 \end{array}$$

for i=1,…,2h.

Consequently, $m\left(f,\Sigma_{2,h}\right)\geq 2$.

In order to prove (ii), we consider:

$$\begin{array}{c} U_{1,h}:={\left(U_{1}^{\left(1\right)},\ldots ,U_{h}^{\left(1\right)},U_{1}^{\left(2\right)},\ldots ,U_{h}^{\left(2\right)}\right)}^{t} \end{array}$$

to be a random vector with independent N(0,1) distributed entries. For i=1,…,h and k=h+1,…,2h such that k−h=i, we obtain:

$$\begin{array}{cc} E\left(U_{1,h}^{\left(i\right)}U_{1,h}^{\left(k\right)}f\left(U_{1,h}\right)\right) & =E\left(U_{i}^{\left(1\right)}U_{k-h}^{\left(2\right)}1_{\left\{\tilde{\Pi }\left(U_{1}^{\left(1\right)},\ldots ,U_{h}^{\left(1\right)}\right)=\tilde{\Pi }\left(U_{1}^{\left(2\right)},\ldots ,U_{h}^{\left(2\right)}\right)\right\}}\right)\\ \; & =\sum_{\pi \in S_{h}}E\left(U_{i}^{\left(1\right)}U_{i}^{\left(2\right)}1_{\left\{\tilde{\Pi }\left(U_{1}^{\left(1\right)},\ldots ,U_{h}^{\left(1\right)}\right)=\tilde{\Pi }\left(U_{1}^{\left(2\right)},\ldots ,U_{h}^{\left(2\right)}\right)=\pi \right\}}\right)\\ \; & =\sum_{\pi \in S_{h}}{\left(E\left(U_{i}^{\left(1\right)}1_{\left\{\tilde{\Pi }\left(U_{1}^{\left(1\right)},\ldots ,U_{h}^{\left(1\right)}\right)=\pi \right\}}\right)\right)}^{2}\\ \; & \neq 0, \end{array}$$

since $E\left(U_{i}^{\left(1\right)}1_{\left\{\tilde{\Pi }\left(U_{1}^{\left(1\right)},\ldots ,U_{h}^{\left(1\right)}\right)=\pi \right\}}\right)\neq 0$ for all $\pi \in S_{h}$. This was shown in the proof of Lemma 3.4 in [20].

All in all, we derive $m(f,\Sigma_{2,h})=2$ and hence, have proven the lemma. □

The case $m(f,\Sigma_{2,h})=2$ exhibits the property that the standard range of the long-range dependence parameter $d^{*}\in \left(0,\frac{1}{2}\right)$ has to be divided into two different sets. If $d^{*}\in \left(\frac{1}{4},\frac{1}{2}\right)$, the transformed process $f{\left(Y_{j,h}\right)}_{j\in Z}$ is still long-range dependent, as can be seen in [16], Table 5.1. If $d^{*}\in \left(0,\frac{1}{4}\right)$, the transformed process is short-range dependent, which means by definition that the autocorrelations of the transformed process are summable, as can be seen in [13], Remark 2.3. Therefore, we have two different asymptotic distributions that have to be considered for the estimator ${\hat{p}}_{n}$ of coincident patterns.

### 3.1. Limit Theorem for the Estimator of *p* in Case of Long-Range Dependence

First, we restrict ourselves to the case that at least one of the two parameters $d_{1}$ and $d_{2}$ is in $\left(\frac{1}{4},\frac{1}{2}\right)$. This assures $d^{*}\in \left(\frac{1}{4},\frac{1}{2}\right)$. We explicitly include mixing cases where the process corresponding to $\min\left\{d_{1},d_{2}\right\}$ is allowed to be long-range as well as short-range dependent.

Note that this setting includes the pure long-range dependence case, which means that for p=1,2, we have $d_{p}\in \left(\frac{1}{4},\frac{1}{2}\right)$, or even $d_{1}=d_{2}=d^{*}$. However, in general, the assumptions are lower, such that we only require $d_{p}\in \left(\frac{1}{4},\frac{1}{2}\right)$ for either p=1 or p=2 and the other parameter is also allowed to be in $\left(-\infty ,0\right)$ or $\left(0,\frac{1}{4}\right)$.

We can, therefore, apply the results of Corollary 1 and obtain the following asymptotic distribution for ${\hat{p}}_{n}$:

**Theorem** **3.** *Under the assumption in **(L)**, we obtain:*

(18) $$\begin{array}{cc} n^{1-2d^{*}}{\left(C_{2}\right)}^{-\frac{1}{2}} & \left({\hat{p}}_{n}-p\right)\overset{D}{\to }\sum_{p,q\in P^{*}}{\tilde{\alpha }}^{\left(p,q\right)}Z_{2,d^{*}+1/2}^{\left(p,q\right)}\left(1\right) \end{array}$$

with $Z_{2,d^{*}+1/2}^{\left(p,q\right)}\left(1\right)$ as given in Theorem 1 for $p,q\in P^{*}$ and $C_{2}:=\frac{1}{2d^{*}\left(4d^{*}-1\right)}$ being a normalizing constant. We have:

$$\begin{array}{c} {\tilde{\alpha }}^{\left(p,q\right)}:=\sum_{i,k=1}^{h}\alpha_{i,k}^{\left(p,q\right)},\;where\;\alpha_{i,k}^{\left(p,q\right)}=\alpha_{i+(p-1)h,k+(q-1)h}, \end{array}$$

for each $p,q\in P^{*}$ and i,k=1,…,h and ${\left(\alpha_{i,k}\right)}_{1\leq i,k\leq dh}=\Sigma_{2,h}^{-1}C\Sigma_{2,h}^{-1}$, where the variable:

$$\begin{array}{c} C={\left(c_{i,k}\right)}_{1\leq i,k\leq 2h}=E\left(Y_{1,h}\left(1_{\left\{\tilde{\Pi }\left(Y_{1}^{\left(1\right)},\ldots ,Y_{h}^{\left(1\right)}\right)=\tilde{\Pi }\left(Y_{1}^{\left(2\right)},\ldots ,Y_{h}^{\left(2\right)}\right)\right\}}-p\right)Y_{1,h}^{t}\right) \end{array}$$

*denotes the matrix of second order Hermite coefficients.*

**Proof.**  The proof of this theorem is an immediate application of the Corollary 1 and Lemma 1. Note that for ${\hat{p}}_{n}$ it holds that it is square integrable with respect to $Y_{j,h}$ and that the set of discontinuity points is a Null set with respect to the 2h-dimensional Lebesgue measure. This is shown in [18], Equation (4.5). □

Following Theorem 2, we are also able to express the limit distribution above in terms of two standard Rosenblatt random variables by modifying the weighting factors in the limit distribution. Note that this requires slightly stronger assumptions as in Theorem 1.

**Theorem** **4.** *Let **(L)** hold with $d_{1}=d_{2}$. Additionally, we assume that $r^{\left(1,1\right)}\left(l\right)=r^{\left(2,2\right)}\left(l\right)$, for l=0,…,h−1, and $L_{1,1}+L_{2,2}\neq L_{1,2}+L_{2,1}$. Then, we obtain:*

$$\begin{array}{cc} n^{1-2d^{*}}{\left(C_{2}\right)}^{-\frac{1}{2}}\left({\hat{p}}_{n}-p\right) & \overset{D}{\to }\left({\tilde{\alpha }}^{\left(1,1\right)}-{\tilde{\alpha }}^{\left(1,2\right)}\right)\frac{L_{2,2}-L_{2,1}-L_{1,2}+L_{1,1}}{2}Z_{2,d^{*}+1/2}^{*}\left(1\right)\\ \; & \;\;+\left({\tilde{\alpha }}^{\left(1,1\right)}+{\tilde{\alpha }}^{\left(1,2\right)}\right)\frac{L_{2,2}+L_{2,1}+L_{1,2}+L_{1,1}}{2}Z_{2,d^{*}+1/2}^{**}\left(1\right), \end{array}$$

with $C_{2}$ and ${\tilde{\alpha }}^{\left(p,q\right)}$ as given in Theorem 3. Note that $Z_{2,d^{*}+1/2}^{*}\left(1\right)$ and $Z_{2,d^{*}+1/2}^{**}\left(1\right)$ are both standard Rosenblatt random variables, whose covariance is given by

(19) $$\begin{array}{c} Cov\left(Z_{2,d^{*}+1/2}^{*}\left(1\right),Z_{2,d^{*}+1/2}^{**}\left(1\right)\right)=\frac{{\left(L_{2,2}-L_{1,1}\right)}^{2}}{{\left(L_{1,1}+L_{2,2}\right)}^{2}-{\left(L_{1,2}+L_{2,1}\right)}^{2}}. \end{array}$$

**Remark** **1.** *Following [18], Corollary 3.14, if additionally $r^{\left(1,1\right)}\left(k\right)=r^{\left(2,2\right)}\left(k\right)$ and $r^{\left(1,2\right)}\left(k\right)=r^{\left(2,1\right)}\left(k\right)$ is fulfilled for all k∈Z, then the two limit random variables following a standard Rosenblatt distribution in Theorem 4 are independent. Note that due to the considerations in [21], Equation (10), we know that the distribution of the sum of two independent standard Rosenblatt random variables is not standard Rosenblatt. However, this yields a computational benefit, as it is possible to efficiently simulate the standard Rosenblatt distribution, for details, as can be seen in [21].*

We turn to an example that deals with the asymptotic variance of the estimator of *p* in Theorem 3 in the case h=1.

**Example** **3.** *We focus on the case h=1 and consider the underlying process ${\left(Y_{j,1}\right)}_{j\in Z}={\left(Y_{j}^{\left(1\right)},Y_{j}^{\left(2\right)}\right)}_{j\in Z}$. It is possible to determine the asymptotic variance depending on the correlation $r^{\left(1,2\right)}\left(0\right)$ between these two increment variables.*

*We start with the calculation of the second order Hermite coefficients in the case π=(1,0). This corresponds to the event $\left\{Y_{j}^{\left(1\right)}\geq 0,Y_{j}^{\left(2\right)}\geq 0\right\}$, which yields:*

$$\begin{array}{c} c_{1,1}^{\pi ,2}=E\left(\left({\left(Y_{j}^{\left(1\right)}\right)}^{2}-1\right)1_{\left\{Y_{j}^{\left(1\right)}\geq 0,Y_{j}^{\left(2\right)}\geq 0\right\}}\right) \end{array}$$

*and:*

$$\begin{array}{c} c_{1,2}^{\pi ,2}=E\left(\left(Y_{j}^{\left(1\right)}Y_{j}^{\left(2\right)}\right)1_{\left\{Y_{j}^{\left(1\right)}\geq 0,Y_{j}^{\left(2\right)}\geq 0\right\}}\right). \end{array}$$

*Due to $r^{\left(1,2\right)}\left(0\right)=r^{\left(2,1\right)}\left(0\right)$, we have $\left(Y_{j}^{\left(1\right)},Y_{j}^{\left(2\right)}\right)\overset{D}{=}\left(Y_{j}^{\left(2\right)},Y_{j}^{\left(1\right)}\right)$ and therefore, $c_{1,1}^{\pi ,2}=c_{2,2}^{\pi ,2}$. We identify the second order Hermite coefficients as the ones already calculated in [20], Example 3.13, although we are considering two consecutive increments of a univariate Gaussian process there. However, since the corresponding values are only determined by the correlation between the Gaussian variables, we can simply replace the autocorrelation at lag 1 by the cross-correlation at lag 0. Hence, we obtain:*

$$\begin{array}{cc} c_{1,1}^{\pi ,2} & =\varphi^{2}\left(0\right)r^{\left(1,2\right)}\left(0\right)\sqrt{1-{\left(r^{\left(1,2\right)}\left(0\right)\right)}^{2}},\\ c_{1,2}^{\pi ,2} & =\varphi^{2}\left(0\right)\sqrt{1-{\left(r^{\left(1,2\right)}\left(0\right)\right)}^{2}}. \end{array}$$

*Recall that the inverse $\Sigma_{2,1}^{-1}={\left(g_{i,j}\right)}_{i,j=1,2}$ of the correlation matrix of $\left(Y_{j}^{\left(1\right)},Y_{j}^{\left(2\right)}\right)$ is given by*

$$\begin{array}{c} \Sigma_{2,1}^{-1}=\frac{1}{1-{\left(r^{\left(1,2\right)}\left(0\right)\right)}^{2}}\left(\begin{array}{ccc} 1 & \; & -r^{(1,2})\left(0\right)\\ -r^{\left(1,2\right)}\left(0\right) & \; & 1 \end{array}\right). \end{array}$$

*By using the formula for ${\tilde{\alpha }}^{\left(p,q\right)}$ obtained in [18], Equation (4.23), we derive:*

$$\begin{array}{cc} {\tilde{\alpha }}_{\pi ,2}^{\left(1,1\right)} & =\alpha_{1,1}^{\pi ,2}=\left(g_{1,1}^{2}+g_{1,2}^{2}\right)c_{1,1}^{\pi ,2}+2g_{1,1}g_{1,2}c_{1,2}^{\pi ,2},\\ {\tilde{\alpha }}_{\pi ,2}^{\left(1,2\right)} & =\alpha_{1,2}^{\pi ,2}=\left(g_{1,1}^{2}+g_{1,2}^{2}\right)c_{1,2}^{\pi ,2}+2g_{1,1}g_{1,2}c_{1,1}^{\pi ,2}. \end{array}$$

*Plugging the second order Hermite coefficients and the entries of the inverse of the covariance matrix depending on $r^{\left(1,2\right)}\left(0\right)$ into the formulas, we arrive at:*

$$\begin{array}{c} {\tilde{\alpha }}_{\pi ,2}^{\left(1,1\right)}=\frac{-\varphi^{2}\left(0\right)r^{\left(1,2\right)}\left(0\right)}{{\left(1-{\left(r^{\left(1,2\right)}\left(0\right)\right)}^{2}\right)}^{1/2}} \end{array}$$

*and:*

$$\begin{array}{c} {\tilde{\alpha }}_{\pi ,2}^{\left(1,2\right)}=\frac{\varphi^{2}\left(0\right)}{{\left(1-{\left(r^{\left(1,2\right)}\left(0\right)\right)}^{2}\right)}^{1/2}}. \end{array}$$

*Therefore, in the case h=1, we obtain the following factors in the limit variance in Theorem 3:*

$$\begin{array}{cc} {\tilde{\alpha }}^{\left(1,1\right)} & ={\tilde{\alpha }}^{\left(2,2\right)}=\frac{-2\varphi^{2}\left(0\right)r^{\left(1,2\right)}\left(0\right)}{{\left(1-{\left(r^{\left(1,2\right)}\left(0\right)\right)}^{2}\right)}^{1/2}}\\ {\tilde{\alpha }}^{\left(1,2\right)} & ={\tilde{\alpha }}^{\left(2,1\right)}=\frac{2\varphi^{2}\left(0\right)}{{\left(1-{\left(r^{\left(1,2\right)}\left(0\right)\right)}^{2}\right)}^{1/2}}. \end{array}$$

**Remark** **2.** *It is not possible to analytically determine the limit variance for h=2, as this includes orthant probabilities of a four-dimensional Gaussian distribution. Following [22], no closed formulas are available for these probabilities. However, there are fast algorithms at hand that calculate the limit variance efficiently. It is possible to take advantage of the symmetry properties of the multivariate Gaussian distribution to keep the computational cost of these algorithms low. For detail, as can be seen in [18], Section 4.3.1.*

### 3.2. Limit Theorem for the Estimator of *p* in Case of Short-Range Dependence

In this section, we focus on the case of $d^{*}\in \left(-\infty ,0\right)\cup \left(0,\frac{1}{4}\right)$. If $d^{*}\in \left(0,\frac{1}{4}\right)$, we are still dealing with a long-range dependent multivariate Gaussian process ${\left(Y_{j,h}\right)}_{j\in Z}$. However, the transformed process ${\hat{p}}_{n}-p$ is no longer long-range dependent, since we are considering a function with Hermite rank 2, see also [16], Table 5.1. Otherwise, if $d^{*}\in \left(-\infty ,0\right)$, the process ${\left(Y_{j,h}\right)}_{j\in Z}$ itself is already short-range dependent, since the cross-correlations are summable. Therefore, we obtain the following central limit theorem by applying Theorem 4 in [14].

**Theorem** **5.** *Under the assumptions in **(S)**, we obtain:*

$$\begin{array}{c} n^{\frac{1}{2}}\left({\hat{p}}_{n}-p\right)\overset{D}{\to }N\left(0,\sigma^{2}\right) \end{array}$$

*with:*

$$\begin{array}{cc} \sigma^{2} & =\sum_{k=-\infty }^{\infty }E[\left(1_{\left\{\tilde{\Pi }\left(Y_{1}^{\left(1\right)},\ldots ,Y_{h}^{\left(1\right)}\right)=\tilde{\Pi }\left(Y_{1}^{\left(2\right)},\ldots ,Y_{h}^{\left(2\right)}\right)\right\}}-p\right)\\ & \;\;\;\;\;\;\times \left(1_{\left\{\tilde{\Pi }\left(Y_{1+k}^{\left(1\right)},\ldots ,Y_{h+k}^{\left(1\right)}\right)=\tilde{\Pi }\left(Y_{1+k}^{\left(2\right)},\ldots ,Y_{h+k}^{\left(2\right)}\right)\right\}}-p\right)]. \end{array}$$

We close this section with a brief retrospect of the results obtained. We established limit theorems for the estimator of *p* as probability of coincident pattern in both time series and hence, on the most important parameter in the context of ordinal pattern dependence. The long-range dependent case as well as the mixed case of short- and long-range dependence was considered. Finally, we provided a central limit theorem for a multivariate Gaussian time series that is short-range dependent if transformed by ${\hat{p}}_{n}$. In the subsequent section, we provide a simulation study that illustrates our theoretical findings. In doing so, we shed light on the Rosenblatt distribution and the distribution of the sum of Rosenblatt distributed random variables.

## 4. Simulation Study

We begin with the generation of a bivariate long-range dependent fractional Gaussian noise series ${\left(Y_{j}^{\left(1\right)},Y_{j}^{\left(2\right)}\right)}_{j=1,\ldots ,n}$.

First, we simulate two independent fractional Gaussian noise processes ${\left(U_{j}^{\left(1\right)}\right)}_{j=1,\ldots ,n}$ and ${\left(U_{j}^{\left(2\right)}\right)}_{j=1,\ldots ,n}$ derived by the R-package “longmemo”, for a fixed parameter $H\in \left(\frac{1}{2},1\right)$ in both time series. For the reader’s convenience, we denote the long-range dependence parameter *d* by $H=d+\frac{1}{2}$ as it is common, when dealing with fractional Gaussian noise and fractional Brownian motion. We refer to *H* as *Hurst parameter*, tracing back to the work of [23]. For H=0.7 and H=0.8 we generate $n=10^{6}$ samples, for H=0.9, we choose $n=2\cdot 10^{6}$. We denote the correlation function of univariate fractional Gaussian noise by $r_{H}^{\left(1,1\right)}\left(k\right)$, k≥0. Then, we obtain ${\left(Y_{j}^{\left(1\right)},Y_{j}^{\left(2\right)}\right)}_{j}$ for j=1,…,n:

(20) $$\begin{array}{cc} \; & Y_{j}^{\left(1\right)}=U_{j}^{\left(1\right)},\\ \; & Y_{j}^{\left(2\right)}=\psi U_{j}^{\left(1\right)}+\phi U_{j}^{\left(2\right)}, \end{array}$$

for ψ,ϕ∈R.

Note that this yields the following properties for the cross-correlations of the two processes for k≥0:

$$\begin{array}{cc} r_{H}^{\left(1,2\right)}\left(k\right) & =E\left(Y_{j}^{\left(1\right)}Y_{j+k}^{\left(2\right)}\right)=\psi r_{H}^{\left(1,1\right)}\left(k\right)\\ r_{H}^{\left(2,1\right)}\left(k\right) & =r^{\left(1,2\right)}\left(-k\right)=\psi r_{H}^{\left(1,1\right)}\left(k\right)\\ r_{H}^{\left(2,2\right)}\left(k\right) & =E\left(Y_{j}^{\left(2\right)}Y_{j+k}^{\left(2\right)}\right)=\left(\psi^{2}+\phi^{2}\right)r_{H}^{\left(1,1\right)}\left(k\right). \end{array}$$

We use ψ=0.6 and ϕ=0.8 to obtain unit variance in the second process.

**Figure 5:**
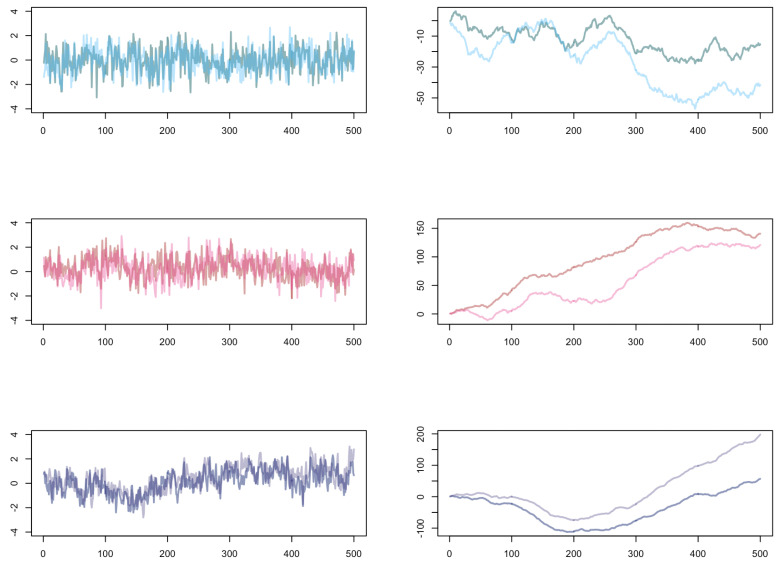
Plots of 500 data points of one path of two dependent fractional Gaussian noise processes (**left**) and the paths of the corresponding fractional Brownian motions (**right**) for different Hurst parameters: H=0.7 (**top**), H=0.8 (**middle**), H=0.9 (**bottom**).

Note that we chose the same Hurst parameter in both processes to get a better simulation result. The simulations of the processes ${\left(Y_{j}^{\left(1\right)}\right)}_{j\in Z}$ and ${\left(Y_{j}^{\left(2\right)}\right)}_{j\in Z}$ are visualized in Figure 5. On the left-hand side, the different fractional Gaussian noises depending on the Hurst parameter *H* are displayed. They represent the stationary long-range dependent Gaussian *increment* processes we need in the view of the limit theorems we derived in Section 3. The processes in which we are comparing the coincident ordinal patterns, namely ${\left(X_{j}^{\left(1\right)}\right)}_{j\in Z}$ and ${\left(X_{j}^{\left(2\right)}\right)}_{j\in Z}$, are shown on the right-hand side in Figure 5. The long-range dependent behavior of the increment processes is very illustrative in these processes: roughly speaking, they become smoother the larger the Hurst parameter gets.

**Figure 6:**
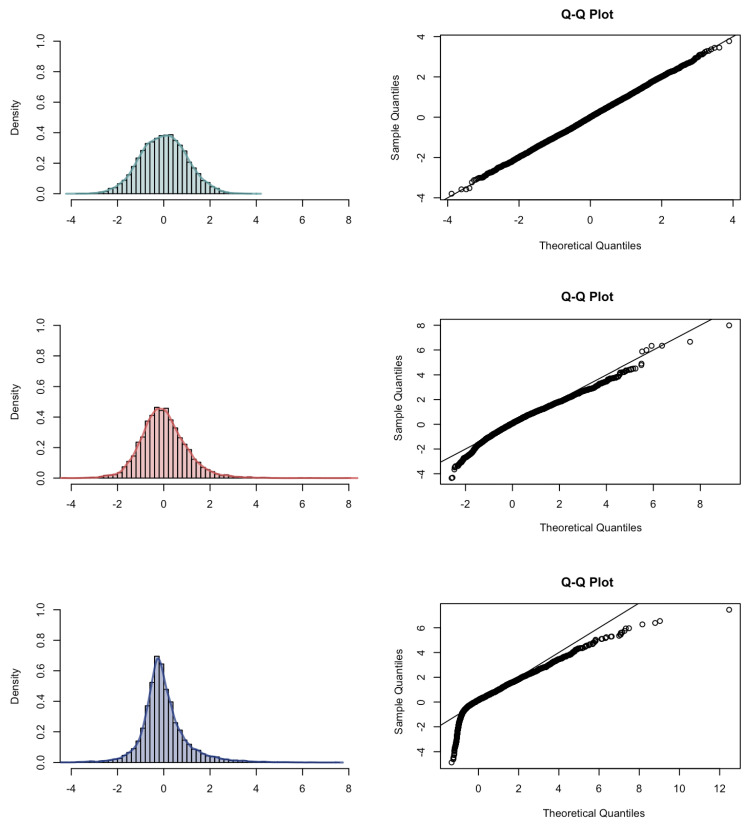
Histogram, kernel density estimation and Q–Q plot with respect to the normal distribution (H=0.7) or to the Rosenblatt distribution of ${\hat{p}}_{n}-p$ with h=2 for different Hurst parameters: H=0.7 (**top**); H=0.8 (**middle**); H=0.9 (**bottom**).

We turn to the simulation results for the asymptotic distribution of the estimator ${\hat{p}}_{n}$. The first limit theorem is given in Theorem 3 for H=0.8 and H=0.9. In the case of H=0.7, a different limit theorem holds, see Theorem 5. Therefore, we turn to the simulation results of the asymptotic distribution of the estimator ${\hat{p}}_{n}$ of *p*, as shown in Figure 6 for pattern length h=2. The asymptotic normality in case H=0.7 can be clearly observed. We turn to the interpretation of the simulation results of the distribution of ${\hat{p}}_{n}-p$ for H=0.8 and H=0.9 as the weighted sum of the sample (cross-)correlations: we observe in the Q–Q plot for H=0.8 that the samples in the upper and lower tail deviate from the reference line. For H=0.9, a similar behavior in the Q–Q plot is observed.

**Figure 7:**
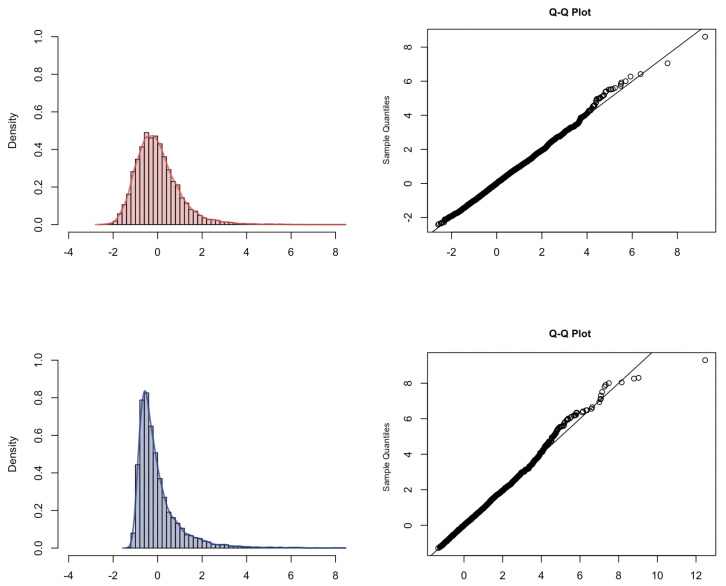
Histogram, kernel density estimation and Q–Q plot with respect to the Rosenblatt distribution of $\frac{1}{n}\sum_{j=1}^{n}H_{2}\left(Y_{j}^{*}\right)$ for different Hurst parameters: H=0.8 (**top**); H=0.9 (**bottom**).

We want to verify the result in Theorem 4 that it is possible, by a different weighting, to express the limit distribution of ${\hat{p}}_{n}-p$ as the distribution of the sum of two independent standard Rosenblatt random variables. The simulated convergence result is provided in Figure 7. We observed the standard Rosenblatt distribution.

## 5. Conclusions and Outlook

We considered limit theorems in the context of the estimation of ordinal pattern dependence in the long-range dependence setting. Pure long-range dependence, as well as mixed cases of short- and long-range dependence, were considered alongside the transformed short-range dependent case. Therefore, we complemented the asymptotic results in [11]. Hence, we made ordinal pattern dependence applicable for long-range dependent data sets as they arise in the context of neurology, as can be seen in [24] or artificial intelligence, as can be seen in [25]. As these kinds of data were already investigated using ordinal patterns, as can be seen, for example, in [26], this emphasizes the large practical impact of the ordinal approach in analyzing the dependence structure multivariate time series. This yields various research opportunities in these fields in the future.

Our results rely on the assumption of Gaussianity of the considered multivariate time series. If we focus on comparing the coincident ordinal patterns in a stationary long-range dependent bivariate time series, we highly benefit from the property of ordinal patterns not being affected by monotone transformations. It is possible to transform the data set to the Gaussian framework without losing the necessary ordinal information. In applications, this property is highly desirable. If we consider the more general setting, that is, stationary increments, the mathematical theory in the background gets a lot more complex leading to the limitations of our results. A crucial argument used in the proofs of the results in Section 2 is given in the *Reduction Theorem*, originally proven in Theorem 4.1 in [27] in the univariate case and extended to the multivariate setting in Theorem 6 in [14]. For further details, we refer the reader to the Appendix A. However, this result only holds in the Gaussian case. Limit theorems for the sample cross-correlation process of multivariate linear long-range dependent processes with Hermite rank 2 have recently been proven in Theorem 4 in [28]. This is possibly an interesting starting point to adapt the proofs in the Appendix A to this larger class of processes without requiring Gaussianity. Considering the property of having a discrete bivariate time series in the background, an interesting extension is given in time continuous processes and the associated techniques of discretization to still regard the ordinal perspective. To think even further beyond our scope, a generalization to categorical data is conceivable and yields an interesting open research opportunity.

## Data Availability

The data that support the findings of this study are available from the corresponding author, Ines Nüßgen, upon reasonable request.

## Appendix A. Technical Appendix

All proofs in this appendix were taken from [18], Chapter 3.

### Appendix A.1. Preliminary Results

Before turning to limit theorems, we introduce a possibility to decompose the *d*-dimensional Gaussian process ${\left(Y_{j}\right)}_{j\in Z}$ using the Cholesky decomposition, as can be seen in [29]. Based on the definition of the multivariate normal distribution, as can be seen in [30], Definition 1.6.1, we find an upper triangular matrix $\tilde{A}$, such that $\tilde{A}{\tilde{A}}^{t}=\Sigma_{d}$. Then, it holds that:

(A1) $$\begin{array}{c} Y_{j}\overset{D}{=}\tilde{A}U_{j}^{*}, \end{array}$$

where $U_{j}^{*}$ is a *d*-dimensional Gaussian process where each $U_{j}^{*}$ has independent and identically N(0,1) distributed entries. We want to assure that ${\left(U_{j}^{*}\right)}_{j\in Z}$ preserves the long-range dependent structure of ${\left(Y_{j}\right)}_{j\in Z}$. Since we know from (2) that:

$$\begin{array}{c} E\left(Y_{j}Y_{j+k}\right)=\Gamma_{Y}\left(k\right)≃k^{D-\frac{1}{2}I_{d}}Lk^{D-\frac{1}{2}I_{d}}\;\left(k\to \infty \right), \end{array}$$

the process $\left(U_{j}^{*}\right)$ has to fulfill:

(A2) $$\begin{array}{c} E\left(U_{j}^{*}U_{j+k}^{*}\right)=\Gamma_{U^{*}}\left(k\right)≃k^{D-\frac{1}{2}I_{d}}L_{U}k^{D-\frac{1}{2}I_{d}}\;\left(k\to \infty \right), \end{array}$$

with $L=\tilde{A}L_{U^{*}}{\tilde{A}}^{t}$.

Then, it holds for all n∈N that:

(A3) $$\begin{array}{c} \left(Y_{j},\;j=1,\ldots ,n\right)\overset{D}{=}\left(\tilde{A}U_{j}^{*},\;j=1,\ldots ,n\right). \end{array}$$

Note that the assumption in (A2) is only well-defined because we assumed $\left|r^{\left(p,q\right)}\left(k\right)\right|<1$ for k≥1 and p,q=1,…,d in (1). This becomes clear in the following considerations. In the proofs of the theorems in this chapter, we do not only need a decomposition of $Y_{j}$, but also of $Y_{j,h}$. As $Y_{j,h}$ is still a multivariate Gaussian process, the covariance matrix of $Y_{j,h}$ given by $\Sigma_{d,h}$ is positive definite. Hence, it is possible to find an upper triangular matrix *A*, such that $AA^{t}=\Sigma_{d,h}$. It holds that:

$$\begin{array}{c} Y_{j,h}\overset{D}{=}AU_{j,h} \end{array}$$

for:

$$\begin{array}{c} U_{j,h}={\left(U_{(j-1)h+1}^{\left(1\right)},\ldots ,U_{jh}^{\left(1\right)},\ldots ,U_{(j-1)h+1}^{\left(d\right)},\ldots ,U_{jh}^{\left(d\right)}\right)}^{t}. \end{array}$$

The random vector $U_{j,h}$ consists of (d·h) independent and standard normally distributed random variables. We notice the different structure of $U_{j,h}$ compared to $Y_{j,h}$. We assure that for consecutive *j*, the entries in $U_{j,h}$ are all different while there are identical entries, for example in $Y_{1,h}={\left(Y_{1}^{\left(1\right)},Y_{2}^{\left(1\right)},\ldots ,Y_{h}^{\left(d\right)}\right)}^{t}$ and $Y_{2,h}={\left(Y_{2}^{\left(1\right)},\ldots ,Y_{h}^{\left(d\right)},Y_{h+1}^{\left(d\right)}\right)}^{t}$. This complicates our aim that:

(A4) $$\begin{array}{c} {\left(Y_{j,h},j=1,\ldots ,n\right)}^{t}\overset{D}{=}{\left(AU_{j,h},\;j=1,\ldots ,n\right)}^{t} \end{array}$$

holds.

The special structure of ${\left(Y_{j,h}\right)}_{j\in Z}$, namely that consisting of *h* consecutive entries of each marginal process $\left(Y_{j}^{\left(p\right)}\right)$, p=1,…,d, alongside the dependence between two random vectors in the process $\left(Y_{j,h}\right)$, has to be reflected in the covariance matrix of $\left(U_{j,h},\;j=1,\ldots ,n\right)$. Hence, we need to check whether such a vector $\left(U_{j,h},\;j=1,\ldots ,n\right)$ exists, i.e., if there is a positive semi-definite matrix that fulfills these conditions. We define A as a block diagonal matrix with *A* as main-diagonal blocks and all off-diagonal blocks as dh×dh-zero matrix.

We denote the covariance matrix of ${\left(Y_{j,h},j=1,\ldots ,n\right)}^{t}$ by $\Sigma_{Y,n}$ and define the following matrix:

(A5) $$\begin{array}{c} \Sigma_{U,n}:=\mathrm{inv}\left(A\right)\Sigma_{Y,n}\mathrm{inv}\left(A^{t}\right). \end{array}$$

We know that $\Sigma_{Y,n}$ is a positive semi-definite for all n∈N because $\left(Y_{j}\right)$ is a Gaussian process. Mathematically described, this means that:

(A6) $$\begin{array}{c} x^{t}\Sigma_{Y,n}x\geq 0, \end{array}$$

for all $x={\left(x_{1},\ldots ,x_{nhd}\right)}^{t}\in R^{nhd}$. We conclude:

$$\begin{array}{cc} x^{t}\Sigma_{U,n}x & \;\;\;=x^{t}\mathrm{inv}\left(A\right)\Sigma_{Y,n}\mathrm{inv}\left(A^{t}\right)x\\ \; & \;\;\;={\left(\mathrm{inv}\left(A^{t}\right)x\right)}^{t}\Sigma_{Y,n}\left(x^{t}\mathrm{inv}\left(A\right)\right)\\ \; & \overset{\left(A6\right)}{\geq }0. \end{array}$$

Therefore, $\Sigma_{U,n}$ is a positive semi-definite matrix for all n∈N and the random vector:

$$\begin{array}{c} {\left(U_{j,h},\;j=1,\ldots ,n\right)}^{t}N∼\left(0,\Sigma_{U,n}\right) \end{array}$$

exists and (A4) holds. Note that we do not have any further information on the dependence structure within the process $\left(U_{j}\right)$, in general, this process neither exhibits long-range dependence, nor independence, nor stationarity.

We continue with two preparatory results that are also necessary for proving Theorem 2.1.

**Lemma** **A1.**
*Let ${\left(Y_{j}\right)}_{j\in Z}$ be a d-dimensional Gaussian process as defined in (1) that fulfills (2) with $d_{1}=\ldots =d_{d}=d^{*}$, such that:*

$$\begin{array}{c} \Gamma_{Y}\left(k\right)=E\left(Y_{j}Y_{j+k}^{t}\right)≃Lk^{2d^{*}-1},\;\left(k\to \infty \right). \end{array}$$

*Let $C_{2}$ be a normalization constant:*

$$\begin{array}{c} C_{2}=\frac{1}{2d^{*}\left(4d^{*}-1\right)} \end{array}$$

*and let $B_{Y}$ be an upper triangular matrix, such that:*

$$\begin{array}{c} B_{Y}B_{Y}^{t}=L. \end{array}$$

*Furthermore, for l∈N we have:*

$$\begin{array}{c} {\hat{\Gamma }}_{Y,n}\left(l\right)=\frac{1}{n-l}\sum_{j=1}^{n-l}Y_{j}Y_{j+l}^{t}. \end{array}$$

*Then, for h∈N it holds that:*

$$\begin{array}{cc} \; & \left(n^{1-2d^{*}}{\left(C_{2}\right)}^{-1/2}{\left(B_{Y}⊗B_{Y}\right)}^{-1}\mathrm{vec}\left({\hat{\Gamma }}_{n}\left(l\right)-\Gamma \left(l\right)\right),\;l=0,\ldots ,h-1\right)\\ \; & \;\;\;\;\;\;\;\;\;\;\;\;\overset{D}{\to }\left(\mathrm{vec}{\left(Z_{2,d^{*}+1/2}^{\left(p,q\right)}\left(1\right)\right)}_{p,q=1,\ldots ,d},\;l=0,\ldots ,h-1\right), \end{array}$$

*where $Z_{2,d^{*}+1/2}^{\left(p,q\right)}\left(1\right)$ has the spectral domain representation:*

$$\begin{array}{c} Z_{2,d^{*}+1/2}^{\left(p,q\right)}\left(1\right)=K_{p,q}\left(d^{*}\right)\int_{R^{2}}^{\prime \prime }\frac{\exp\left(i\left(\lambda_{1}+\lambda_{2}\right)\right)-1}{i\left(\lambda_{1}+\lambda_{2}\right)}{\left|\lambda_{1}\lambda_{2}\right|}^{-d^{*}}{\tilde{B}}^{\left(p\right)}\left(d\lambda_{1}\right){\tilde{B}}^{\left(q\right)}\left(d\lambda_{2}\right) \end{array}$$

*where:*

$$\begin{array}{c} K_{p,q}^{2}\left(d^{*}\right)=\left\{\begin{array}{cc} & \frac{1}{2C_{2}{\left(2\Gamma \left(1-2d^{*}\right)\sin\left(\pi d^{*}\right)\right)}^{2}},\;p=q\\ \; & \frac{1}{C_{2}{\left(2\Gamma \left(1-2d^{*}\right)\sin\left(\pi d^{*}\right)\right)}^{2}},\;\;\;p\neq q. \end{array}\right. \end{array}$$

*and $\tilde{B}\left(d\lambda \right)=\left({\tilde{B}}^{\left(1\right)}\left(d\lambda \right),\ldots ,{\tilde{B}}^{\left(d\right)}\left(d\lambda \right)\right)$ is a multivariate Hermitian–Gaussian random measure as defined in (12).*

**Proof.** First, we can use (A1):

$$\begin{array}{c} Y_{j}\overset{D}{=}\tilde{A}U_{j}^{*}, \end{array}$$

such that $\left(U_{j}^{*}\right)$ is a multivariate Gaussian process with $U_{j}^{*}∼N\left(0,I_{d}\right)$ and $\left(U_{j}^{*}\right)$ is still long-range dependent, as can be seen in (A2). It is possible to decompose the sample cross-covariance matrix ${\hat{\Gamma }}_{Y,n}\left(l\right)-\Gamma_{Y}\left(l\right)$ with respect to $\left(Y_{j}\right)$ at lag *l* given by

$$\begin{array}{c} {\hat{\Gamma }}_{Y,n}\left(l\right)-\Gamma_{Y}\left(l\right)=\frac{1}{n-l}\sum_{j=1}^{n-l}Y_{j}Y_{j+l}^{t}-E\left(Y_{j}Y_{j+l}^{t}\right) \end{array}$$

to:

$$\begin{array}{c} {\hat{\Gamma }}_{Y,n}\left(l\right)-\Gamma_{Y}\left(l\right)\overset{D}{=}\tilde{A}\left({\hat{\Gamma }}_{U^{*},n}\left(l\right)-\Gamma_{U^{*}}\left(l\right)\right){\tilde{A}}^{t}, \end{array}$$

where we define the sample cross-covariance matrix ${\hat{\Gamma }}_{U^{*},n}\left(l\right)-\Gamma_{U^{*}}\left(l\right)$ with respect to $\left(U_{j}^{*}\right)$ at lag *l* by

$$\begin{array}{c} {\hat{\Gamma }}_{U^{*},n}\left(l\right)-\Gamma_{U^{*}}\left(l\right)=\frac{1}{n-l}\sum_{j=1}^{n-l}U_{j}^{*}U_{j+l}^{*}-E\left(U_{j}^{*}U_{j+l}^{*}\right). \end{array}$$

Each entry of:

$$\begin{array}{c} {\hat{\Gamma }}_{U^{*},n}\left(l\right)-\Gamma_{U^{*}}\left(l\right)={\left({\hat{r}}_{n,U^{*}}^{\left(p,q\right)}\left(l\right)-r_{U^{*}}^{\left(p,q\right)}\left(l\right)\right)}_{p,q=1,\ldots ,d} \end{array}$$

is given by

$$\begin{array}{c} {\hat{r}}_{n,U^{*}}^{\left(p,q\right)}\left(l\right)-r_{U^{*}}^{\left(p,q\right)}\left(l\right):=\sum_{j=1}^{n}U_{j}^{*\;(p)}U_{j+l}^{*\;(q)}-E\left(U_{j}^{*\;(p)}U_{j+l}^{*\;(q)}\right). \end{array}$$

Following [31], proof of Lemma 7.4, the limit distribution of:

$$\begin{array}{c} \left({\hat{\Gamma }}_{U^{*},n}\left(l\right)-\Gamma_{U^{*}}\left(l\right),\;l=0,\ldots ,h-1\right) \end{array}$$

is equal to the limit distribution of:

$$\begin{array}{c} \left({\hat{\Gamma }}_{U^{*},n}\left(0\right)-\Gamma_{U^{*}}\left(0\right),\;l=0,\ldots ,h-1\right). \end{array}$$

We recall the assumption that $d^{*}=d_{p}$ for all p=1,…,d. We follow [14], Theorem 6 and use the Cramer–Wold device: Let $a_{1,1},a_{1,2},\ldots ,a_{d,d}\in R$. We are interested in the asymptotic behavior of:

$$\begin{array}{cc} \; & n^{1-2d^{*}}\sum_{p,q=1}^{d}a_{p,q}\left({\hat{r}}_{n,U}^{\left(p,q\right)}\left(0\right)-r_{U}^{\left(p,q\right)}\left(0\right)\right)\\ = & n^{-2d^{*}}\sum_{j=1}^{n}\sum_{p,q=1}^{d}a_{p,q}\left(U_{j}^{*\;(p)}U_{j}^{*\;(q)}-E\left(U_{j}^{*\;(p)}U_{j}^{*\;(q)}\right)\right). \end{array}$$

We consider the function:

(A7) $$\begin{array}{c} f\left(U_{j}^{*}\right)=\sum_{p,q=1}^{d}a_{p,q}\left(U_{j}^{*\;(p)}U_{j}^{*\;(q)}-E\left(U_{j}^{*\;(p)}U_{j}^{*\;(q)}\right)\right) \end{array}$$

and may apply Theorem 6 in [14]. Using the Hermite decomposition of *f* as given in (11), we observe that *f* and therefore, $a_{p,q}$, p,q=1,…,d, only affects the Hermite coefficients. Indeed, using [15], Lemma 3.5, the Hermite coefficients reduce to $a_{p,q}$ for each summand on the right-hand side in (A7). Hence, we can state:

(A8) $$\begin{array}{cc} n^{-2d^{*}}\sum_{j=1}^{n}\sum_{p,q=1}^{d}a_{p,q} & \left(U_{j}^{*\;(p)}U_{j}^{*\;(q)}-E\left(U_{j}^{*\;(p)}U_{j}^{*\;(q)}\right)\right) \end{array}$$

(A9) $$\begin{array}{cc} \; & \overset{D}{\to }\sum_{p,q=1}^{d}a_{p,q}Z_{2,d^{*}+1/2}^{\left(p,q\right)}\left(1\right), \end{array}$$

where $Z_{2,d^{*}+1/2}^{\left(p,q\right)}\left(1\right)$ has the spectral domain representation:

(A10) $$\begin{array}{c} Z_{2,d^{*}+1/2}^{\left(p,q\right)}\left(1\right)=K_{p,q}\left(d^{*}\right)\int_{R^{2}}^{\prime \prime }\frac{\exp\left(i\left(\lambda_{1}+\lambda_{2}\right)\right)-1}{i\left(\lambda_{1}+\lambda_{2}\right)}{\left|\lambda_{1}\lambda_{2}\right|}^{-d^{*}}{\tilde{B}}^{\left(p\right)}\left(d\lambda_{1}\right){\tilde{B}}^{\left(q\right)}\left(d\lambda_{2}\right) \end{array}$$

where:

$$\begin{array}{c} K_{p,q}^{2}\left(d^{*}\right)=\left\{\begin{array}{cc} & \frac{1}{2C_{2}{\left(2\Gamma \left(1-2d^{*}\right)\sin\left(\pi d^{*}\right)\right)}^{2}},\;p=q\\ \; & \frac{1}{C_{2}{\left(2\Gamma \left(1-2d^{*}\right)\sin\left(\pi d^{*}\right)\right)}^{2}},\;\;p\neq q. \end{array}\right. \end{array}$$

and $\tilde{B}\left(d\lambda \right)=\left({\tilde{B}}^{\left(1\right)}\left(d\lambda \right),\ldots ,{\tilde{B}}^{\left(d\right)}\left(d\lambda \right)\right)$ is an appropriate multivariate Hermitian–Gaussian random measure. Thus, we proved convergence in the distribution of the sample-cross correlation matrix:

$$\begin{array}{c} n^{1-2d^{*}}\left({\hat{\Gamma }}_{U^{*},n}\left(0\right)-\Gamma_{U^{*}}\left(0\right)\right)\overset{D}{\to }{\left(Z_{2,d^{*}+1/2}^{\left(p,q\right)}\left(1\right)\right)}_{p,q=1,\ldots ,d}. \end{array}$$

We take a closer look at the covariance matrix of $\mathrm{vec}\left({\hat{\Gamma }}_{U^{*},n}\left(0\right)-\Gamma_{U^{*}}\left(0\right)\right)$. Following [28], Lemma 5.7, we observe:

$$\begin{array}{cc} \; & n^{1-2d^{*}}{\left(4d^{*}\left(4d^{*}-1\right)\right)}^{1/2}\mathrm{Cov}\left(\mathrm{vec}\left({\hat{\Gamma }}_{U^{*},n}\left(0\right)-\Gamma_{U^{*}}\left(0\right)\right),\mathrm{vec}\left({\hat{\Gamma }}_{U^{*},n}\left(0\right)-\Gamma_{U^{*}}\left(0\right)\right)\right)\\ = & \left(I_{d^{2}}+K_{d^{2}}\right)\left(L_{U^{*}}⊗L_{U^{*}}\right), \end{array}$$

with $L_{U^{*}}$ as defined in (A3) and ⊗ denotes the Kronecker product. Furthermore, $K_{d}$ denotes the commutation matrix that that transforms vec(A) into $\mathrm{vec}\left(A^{t}\right)$ for $A\in R^{d\times d}$. Further details can be found in [32]. Hence, the covariance matrix of the vector of the sample cross-covariances is fully specified by the knowledge of $L_{U^{*}}$ as it arises in the context of long-range dependence in (A3). We obtain a relation between *L* and $L_{U^{*}}$, since:

$$\begin{array}{c} \Gamma_{Y}\left(\cdot \right)=\tilde{A}\Gamma_{U}\left(\cdot \right){\tilde{A}}^{t}. \end{array}$$

Both:

$$\begin{array}{c} \Gamma_{Y}\left(k\right)≃Lk^{2d^{*}-1}\;\left(k\to \infty \right) \end{array}$$

and:

$$\begin{array}{c} \Gamma_{U^{*}}\left(k\right)≃L_{U^{*}}k^{2d^{*}-1}\;\left(k\to \infty \right) \end{array}$$

hold and we obtain:

$$\begin{array}{c} L=\tilde{A}L_{U^{*}}{\tilde{A}}^{t}. \end{array}$$

We study the covariance matrix of: $\mathrm{vec}\left({\hat{\Gamma }}_{Y,n}\left(0\right)-\Gamma_{Y}\left(0\right)\right)$:

(A11) $$\begin{array}{cc} \; & n^{1-2d^{*}}{\left(4d^{*}\left(4d^{*}-1\right)\right)}^{1/2}\mathrm{Cov}\left(\mathrm{vec}\left({\hat{\Gamma }}_{Y,n}\left(0\right)-\Gamma_{Y}\left(0\right)\right),\mathrm{vec}{\left({\hat{\Gamma }}_{Y,n}\left(0\right)-\Gamma_{Y}\left(0\right)\right)}^{t}\right)\\ \to & \;\left(I_{d^{2}}+K_{d^{2}}\right)\left(L⊗L\right)\\ = & \;\left(I_{d^{2}}+K_{d^{2}}\right)\left(\tilde{A}L_{U^{*}}{\tilde{A}}^{t}\right)⊗\left(\tilde{A}L_{U^{*}}{\tilde{A}}^{t}\right)\\ = & \;\left(I_{d^{2}}+K_{d^{2}}\right)\left(\tilde{A}⊗\tilde{A}\right)\cdot \left(L_{U^{*}}⊗L_{U^{*}}\right)\cdot \left({\tilde{A}}^{t}⊗{\tilde{A}}^{t}\right). \end{array}$$

Let $B_{U^{*}}$ be an upper triangular matrix, such that:

$$\begin{array}{c} B_{U^{*}}B_{U^{*}}^{t}:=L_{U^{*}}. \end{array}$$

We know that such a matrix exists because $L_{U^{*}}$ is positive definite. Analogously, we define $B_{Y}$:

$$\begin{array}{c} B_{Y}:=\tilde{A}B_{U^{*}}. \end{array}$$

Then, it holds that:

$$\begin{array}{c} B_{Y}B_{Y}^{t}=L. \end{array}$$

We arrive at:

$$\begin{array}{cc} \; & n^{1-2d^{*}}{\left(C_{2}\right)}^{-1/2}{\left(B_{Y}⊗B_{Y}\right)}^{-1}\mathrm{vec}\left({\hat{\Gamma }}_{Y,n}\left(0\right)-\Gamma_{Y}\left(0\right)\right)\\ \; & \overset{D}{=}n^{1-2d^{*}}{\left(C_{2}\right)}^{-1/2}{\left(B_{U^{*}}⊗B_{U^{*}}\right)}^{-1}{\left(A⊗A\right)}^{-1}\mathrm{vec}\left(\tilde{A}\left({\hat{\Gamma }}_{U^{*},n}\left(0\right)-\Gamma_{U^{*}}\left(0\right)\right){\tilde{A}}^{t}\right)\\ \; & =n^{1-2d^{*}}{\left(C_{2}\right)}^{-1/2}{\left(B_{U^{*}}⊗B_{U^{*}}\right)}^{-1}\mathrm{vec}\left({\hat{\Gamma }}_{U^{*},n}\left(0\right)-\Gamma_{U^{*}}\left(0\right)\right)\\ \; & \overset{D}{\to }\mathrm{vec}{\left(Z_{2,d^{*}+1/2}^{\left(p,q\right)}\left(1\right)\right)}_{p,q=1,\ldots ,d}, \end{array}$$

where $Z_{2,d^{*}+1/2}^{\left(p,q\right)}\left(1\right)$ has the spectral domain representation:

$$\begin{array}{c} Z_{2,d^{*}+1/2}^{\left(p,q\right)}\left(1\right)=K_{p,q}\left(d^{*}\right)\int_{R^{2}}^{\prime \prime }\frac{\exp\left(i\left(\lambda_{1}+\lambda_{2}\right)\right)-1}{i\left(\lambda_{1}+\lambda_{2}\right)}{\left|\lambda_{1}\lambda_{2}\right|}^{-d^{*}}{\tilde{B}}^{\left(p\right)}\left(d\lambda_{1}\right){\tilde{B}}^{\left(q\right)}\left(d\lambda_{2}\right) \end{array}$$

where:

$$\begin{array}{c} K_{p,q}^{2}\left(d^{*}\right)=\left\{\begin{array}{cc} & \frac{1}{2C_{2}{\left(2\Gamma \left(1-2d^{*}\right)\sin\left(\pi d^{*}\right)\right)}^{2}},\;p=q\\ \; & \frac{1}{C_{2}{\left(2\Gamma \left(1-2d^{*}\right)\sin\left(\pi d^{*}\right)\right)}^{2}},\;\;\;p\neq q. \end{array}\right. \end{array}$$

and $\tilde{B}\left(d\lambda \right)=\left({\tilde{B}}^{\left(1\right)}\left(d\lambda \right),\ldots ,{\tilde{B}}^{\left(d\right)}\left(d\lambda \right)\right)$ is a multivariate Hermitian–Gaussian random measure as defined in (12). Note that the standardization on the left-hand side is appropriate since the covariance matrix of $\mathrm{vec}\left(Z_{2,d^{*}+1/2}\left(1\right)\right)$ is given by

(A12) $$\begin{array}{cc} E(K^{2}\left(d^{*}\right)\int_{R^{2}}^{\prime \prime } & \int_{R^{2}}^{\prime \prime }E_{\lambda_{1},\lambda_{2}}\bar{E_{\lambda_{3},\lambda_{4}}}\mathrm{vec}\left(\tilde{B}\left(d\lambda_{1}\right){\left(\tilde{B}\left(d\lambda_{2}\right)\right)}^{t}\right)\\ \; & \;\;\;\;\;\;\;\;\;{\left(\mathrm{vec}\left(\bar{\tilde{B}\left(d\lambda_{3}\right){\left(\tilde{B}\left(d\lambda_{4}\right)\right)}^{t}}\right)\right)}^{t}). \end{array}$$

by denoting:

$$\begin{array}{c} E_{\lambda_{1},\lambda_{2}}:=\frac{\exp\left(i\left(\lambda_{1}+\lambda_{2}\right)\right)-1}{i\left(\lambda_{1}+\lambda_{2}\right)}{\left|\lambda_{1}\lambda_{2}\right|}^{-d^{*}}. \end{array}$$

We observe:

(A13) $$\begin{array}{cc} \; & E\left(\mathrm{vec}\left(\tilde{B}\left(d\lambda_{1}\right)\tilde{B}{\left(d\lambda_{2}\right)}^{t}\right){\left(\mathrm{vec}\left(\bar{\tilde{B}\left(d\lambda_{3}\right){\left(\tilde{B}\left(d\lambda_{4}\right)\right)}^{t}}\right)\right)}^{t}\right)\\ = & \left\{\begin{array}{cc} \; & I_{d^{2}}d\lambda_{1}d\lambda_{2},\;\left|\lambda_{1}\right|=\left|\lambda_{3}\right|\neq \left|\lambda_{2}\right|=\left|\lambda_{4}\right|,\\ \; & K_{d^{2}}d\lambda_{1}d\lambda_{2},\;\left|\lambda_{1}\right|=\left|\lambda_{4}\right|\neq \left|\lambda_{2}\right|=\left|\lambda_{3}\right|, \end{array}\right. \end{array}$$

following [28], (27). Neither the case $\left|\lambda_{1}\right|=\left|\lambda_{2}\right|$ nor $\left|\lambda_{3}\right|=\left|\lambda_{4}\right|$ has to be incorporated as the diagonals are excluded in the integration in (A12). □

**Corollary** **A1.**
*Under the assumptions of Lemma A1, there is a different representation of the limit random vector. For h∈N, we obtain:*

$$\begin{array}{c} \left(n^{1-2d^{*}}{\left(C_{2}\right)}^{-1/2}\mathrm{vec}\left({\hat{\Gamma }}_{n}\left(l\right)-\Gamma \left(l\right)\right),\;l=0,\ldots ,h-1\right)\overset{D}{\to }\left(\mathrm{vec}\left(Z_{2,d^{*}+1/2}\left(1\right)\right)\;l=0,\ldots ,h-1\right), \end{array}$$

*where $\mathrm{vec}\left(Z_{2,d^{*}+1/2}\left(1\right)\right)$ has the spectral domain representation:*

$$\begin{array}{c} \mathrm{vec}\left(Z_{2,d^{*}+1/2}\left(1\right)\right)=D_{K\left(d^{*}\right)}\int_{R^{2}}^{\prime \prime }\frac{\exp\left(i\left(\lambda_{1}+\lambda_{2}\right)\right)-1}{i\left(\lambda_{1}+\lambda_{2}\right)}{\left|\lambda_{1}\lambda_{2}\right|}^{-d^{*}}\mathrm{vec}\left({\tilde{B}}_{L}\left(d\lambda_{1}\right){\tilde{B}}_{L}{\left(d\lambda_{2}\right)}^{t}\right). \end{array}$$

*The matrix $D_{K\left(d^{*}\right)}$ is a diagonal matrix:*

$$\begin{array}{c} D_{K\left(d^{*}\right)}=\mathrm{diag}\left(\mathrm{vec}\left(K\left(d^{*}\right)\right)\right), \end{array}$$

*and $K\left(d^{*}\right)={\left(K_{p,q}\left(d^{*}\right)\right)}_{p,q=1,\ldots ,d}$ is such that:*

$$\begin{array}{c} K^{2}{\left(d^{*}\right)}_{p,q}=\left\{\begin{array}{cc} & \frac{1}{2C_{2}{\left(2\Gamma \left(1-2d^{*}\right)\sin\left(\pi d^{*}\right)\right)}^{2}},\;p=q\\ \; & \frac{1}{C_{2}{\left(2\Gamma \left(1-2d^{*}\right)\sin\left(\pi d^{*}\right)\right)}^{2}},\;\;\;p\neq q. \end{array}\right. \end{array}$$

*Furthermore, ${\tilde{B}}_{L}\left(d\lambda \right)$ is a multivariate Hermitian–Gaussian random measure that fulfills:*

$$\begin{array}{c} E\left({\tilde{B}}_{L}\left(d\lambda \right){\tilde{B}}_{L}{\left(d\lambda \right)}^{*}\right)=L\;d\lambda . \end{array}$$

**Proof.** The proof is an immediate consequence of Lemma A1 using ${\tilde{B}}_{L}\left(d\lambda \right)=B_{Y}\tilde{B}\left(d\lambda \right)$ with $B_{Y}B_{Y}^{t}=L$ and $\tilde{B}\left(d\lambda \right)$ as defined in (12). □

### Appendix A.2. Proof of Theorem 2.1

**Proof.** Without loss of generality, we assume $E\left(f\left(Y_{j,h}\right)\right)=0$. Following the argumentation in [20], Theorem 5.9, we first remark that $Y_{j,h}\overset{D}{=}AU_{j,h}$ with $U_{j,h}$ and *A* as described in (A4) and (A5). We now want to study the asymptotic behavior of the partial sum $\sum_{j=1}^{n}f^{*}\left(U_{j}\right)$ where $f^{*}\left(U_{j,h}\right):=f\left(AU_{j,h}\right)\overset{D}{=}f\left(Y_{j,h}\right)$. Since $m\left(f^{*},I_{dh}\right)=m\left(f\circ A,I_{dh}\right)=m\left(f,\Sigma_{d,h}\right)=2$, as can be seen in [15], Lemma 3.7, hence, we know by [14], Theorem 6, that these partial sums are dominated by the second order terms in the Hermite expansion of $f^{*}$:

$$\begin{array}{cc} \sum_{j=1}^{n}f^{*}\left(U_{j,h}\right) & \sum_{j=1}^{n}\sum_{l_{1}+\ldots +l_{dh}=2}E\left(f^{*}\left(U_{j,h}\right)H_{l_{1},\ldots ,l_{dh}}\left(U_{j,h}\right)\right)H_{l_{1},\ldots ,l_{dh}}\left(U_{j,h}\right)+o_{P}\left(n^{2d^{*}}\right). \end{array}$$

This follows from the multivariate extension of the Reduction Theorem as proven in [14]. We obtain:

$$\begin{array}{cc} \; & \sum_{l_{1}+\ldots +l_{dh}=2}E\left(f^{*}\left(U_{j,h}\right)H_{l_{1},\ldots ,l_{dh}}\left(U_{j,h}\right)\right)H_{l_{1},\ldots ,l_{dh}}\left(U_{j,h}\right)\\ = & \sum_{i=1}^{dh}E\left(f^{*}\left(U_{j,h}\right)\left({\left(U_{j,h}^{\left(i\right)}\right)}^{2}-1\right)\right)\left({\left(U_{j,h}^{\left(i\right)}\right)}^{2}-1\right)+\sum_{1\leq i,k\leq dh,i\neq k}E\left(f^{*}\left(U_{j,h}\right)U_{j,h}^{\left(i\right)}U_{j,h}^{\left(k\right)}\right)U_{j,h}^{\left(i\right)}U_{j,h}^{\left(k\right)}\\ = & \sum_{i=1}^{dh}E\left(f^{*}\left(U_{j,h}\right){\left(U_{j,h}^{\left(i\right)}\right)}^{2}\right)\left({\left(U_{j,h}^{\left(i\right)}\right)}^{2}-1\right)+\sum_{1\leq i,k\leq dh,i\neq k}E\left(f^{*}\left(U_{j,h}\right)U_{j,h}^{\left(i\right)}U_{j,h}^{\left(k\right)}\right)U_{j,h}^{\left(i\right)}U_{j,h}^{\left(k\right)}, \end{array}$$

since $E\left(f^{*}\left(U_{j,h}\right)\right)=E\left(f\left(Y_{j,h}\right)\right)=0$. This results in:

(A14) $$\begin{array}{cc} \; & \sum_{i=1}^{dh}E\left(f^{*}\left(U_{j,h}\right){\left(U_{j,h}^{\left(i\right)}\right)}^{2}\right)\left({\left(U_{j,h}^{\left(i\right)}\right)}^{2}-1\right)+\sum_{1\leq i,k\leq dh,i\neq k}E\left(f^{*}\left(U_{j,h}\right)U_{j,h}^{\left(i\right)}U_{j,h}^{\left(k\right)}\right)U_{j,h}^{\left(i\right)}U_{j,h}^{\left(k\right)}\\ = & \sum_{1\leq i,k\leq dh}E\left(f^{*}\left(U_{j,h}\right)U_{j,h}^{\left(i\right)}U_{j,h}^{\left(k\right)}\right)U_{j,h}^{\left(i\right)}U_{j,h}^{\left(k\right)}-\sum_{i=1}^{dh}E\left(f^{*}\left(U_{j,h}\right){\left(U_{j,h}^{\left(i\right)}\right)}^{2}\right). \end{array}$$

Note that:

(A15) $$\begin{array}{c} \sum_{1\leq i,k\leq dh}E\left(f^{*}\left(U_{j,h}\right)U_{j,h}^{\left(i\right)}U_{j,h}^{\left(k\right)}\right)E\left(U_{j,h}^{\left(i\right)}U_{j,h}^{\left(k\right)}\right)=\sum_{i=1}^{dh}E\left(f^{*}\left(U_{j,h}\right){\left(U_{j,h}^{\left(i\right)}\right)}^{2}\right) \end{array}$$

since the entries of $U_{j,h}$ are independent for fixed *j* and identically N(0,1) distributed. Thus, the subtrahend in (A14) equals the expected value of the minuend. Define $B:={\left(b_{i,k}\right)}_{1\leq i,k\leq dh}\in R^{\left(dh)\times (dh\right)}$ with:

$$\begin{array}{c} b_{i,k}:=E\left(f^{*}\left(U_{j,h}\right)U_{j,h}^{\left(i\right)}U_{j,h}^{\left(k\right)}\right)=E\left(f^{*}\left(U_{1}\right)U_{1}^{\left(i\right)}U_{1}^{\left(k\right)}\right) \end{array}$$

since we are considering a stationary process. We obtain:

$$\begin{array}{c} B=E\left(U_{j,h}f^{*}\left(U_{j,h}\right)U_{j,h}^{t}\right)=E\left(A^{-1}Y_{j,h}f\left(Y_{j,h}\right)Y_{j,h}^{t}{\left(A^{-1}\right)}^{t}\right). \end{array}$$

Hence, we can state the following:

(A16) $$\begin{array}{cc} \; & \sum_{1\leq i,k\leq dh}E\left(f^{*}\left(U_{j,h}\right)U_{j,h}^{\left(i\right)}U_{j,h}^{\left(k\right)}\right)U_{j,h}^{\left(i\right)}U_{j,h}^{\left(k\right)}\\ \; & =U_{j,h}^{t}BU_{j,h}\\ \; & \overset{D}{=}Y_{j,h}^{t}{\left(A^{-1}\right)}^{t}BA^{-1}Y_{j,h}\\ \; & =Y_{j,h}^{t}{\left(A^{-1}\right)}^{t}A^{-1}E\left(Y_{j,h}f\left(Y_{j,h}\right)Y_{j,h}^{t}\right){\left(A^{-1}\right)}^{t}A^{-1}Y_{j,h}\\ \; & =Y_{j,h}^{t}\Sigma_{d,h}^{-1}E\left(Y_{j,h}f\left(Y_{j,h}\right)Y_{j,h}^{t}\right)\Sigma_{d,h}^{-1}Y_{j,h}\\ \; & =Y_{j,h}^{t}AY_{j,h}\\ \; & =\sum_{1\leq i,k\leq dh}Y_{j}^{\left(i\right)}Y_{j}^{\left(k\right)}\alpha_{ik}, \end{array}$$

where we define $A:={\left(\alpha_{ik}\right)}_{1\leq ik\leq dh}:=\Sigma_{d,h}^{-1}C\Sigma_{d,h}^{-1}$, with $C:=E\left(Y_{j,h}f\left(Y_{j,h}\right)Y_{j,h}^{t}\right)$ as the matrix of second order Hermite coefficients, in contrast to *B* now with respect to the original considered process ${\left(Y_{j,h}\right)}_{j\in Z}$. Remembering the special structure of $Y_{j,h}={\left(Y_{j}^{\left(1\right)},\ldots ,Y_{j+h-1}^{\left(1\right)},\ldots ,Y_{j}^{\left(d\right)},Y_{j+h-1}^{\left(d\right)}\right)}^{t}$, namely that $Y_{j,h}^{\left(k\right)}=Y_{j+(k\;\mathrm{mod}\;h)-1}^{\left(\lfloor \frac{k-1}{h}\rfloor +1\right)}$, k=1,…,dh, we can see that:

(A17) $$\begin{array}{cc} \sum_{j=1}^{n}\sum_{1\leq ik\leq dh}Y_{j,h}^{\left(i\right)}Y_{j,h}^{\left(k\right)}\alpha_{ik}\; & =\sum_{j=1}^{n}\sum_{1\leq ik\leq dh}Y_{j+(i\;\mathrm{mod}\;h)-1}^{\left(\left\lfloor \frac{i-1}{h}\right\rfloor +1\right)}Y_{j+(k\;\mathrm{mod}\;h)-1}^{\left(\left\lfloor \frac{k-1}{h}\right\rfloor +1\right)}\alpha_{ik}\\ \; & =\sum_{j=1}^{n}\sum_{p,q=1}^{d}\sum_{i,k=1}^{h}Y_{j+i-1}^{\left(p\right)}Y_{j+k-1}^{\left(q\right)}\alpha_{ik}^{\left(p,q\right)}, \end{array}$$

where we divide:

$$\begin{array}{c} A=\left(\begin{array}{ccccccc} A^{\left(1,1\right)} & \; & A^{\left(1,2\right)} & \; & \ldots & \; & A^{\left(1,d\right)}\\ A^{\left(2,1\right)} & \; & A^{\left(2,2\right)} & \; & \ldots & \; & A^{\left(2,d\right)}\\ \vdots & \; & \vdots & \; & \vdots \\ A^{\left(d,1\right)} & \; & A^{\left(d,2\right)} & \; & \ldots & \; & A^{\left(d,d\right)} \end{array}\right), \end{array}$$

with $A^{\left(p,q\right)}={\left(\alpha_{i,k}^{\left(p,q\right)}\right)}_{1\leq i,k\leq h}\in R^{h\times h}$ such that $\alpha_{i,k}^{\left(p,q\right)}=\alpha_{i+(p-1)h,k+(q-1)h}$ for each p,q=1,…,d and i,k=1,…,h. We can now split the considered sum in (A17) in a way such that we are afterwards able to express it in terms of sample cross-covariances. In order to do so, we define the sample cross-covariance at lag *l* by

$$\begin{array}{c} {\hat{r}}_{n}^{\left(p,q\right)}\left(l\right):=\frac{1}{n}\sum_{j=1}^{n-l}X_{j}^{\left(p\right)}X_{j+l}^{\left(q\right)} \end{array}$$

for p,q=1,…,d. Note that in the case h=1, it follows directly that:

$$\begin{array}{c} \sum_{j=1}^{n}\sum_{p,q=1}^{d}\sum_{i,k=1}^{h}Y_{j+i-1}^{\left(p\right)}Y_{j+k-1}^{\left(q\right)}\alpha_{ik}^{\left(p,q\right)}=\sum_{p,q=1}^{d}\alpha_{1,1}^{\left(p,q\right)}\sum_{j=1}^{n}Y_{j}^{\left(p\right)}Y_{j}^{\left(q\right)}=n\sum_{p,q=1}^{d}{\hat{r}}_{n}^{\left(p,q\right)}\left(0\right). \end{array}$$

The case h=2 has to be regarded separately, too, and we obtain:

$$\begin{array}{cc} \; & \sum_{j=1}^{n}\sum_{p,q=1}^{d}\sum_{i,k=1}^{2}Y_{j+i-1}^{\left(p\right)}Y_{j+k-1}^{\left(q\right)}\alpha_{ik}^{\left(p,q\right)}\\ = & \sum_{p,q=1}^{d}\left(\alpha_{1,1}^{\left(p,q\right)}\sum_{j=1}^{n}Y_{j}^{\left(p\right)}Y_{j}^{\left(q\right)}+\alpha_{1,2}^{\left(p,q\right)}\sum_{j=1}^{n}Y_{j}^{\left(p\right)}Y_{j+1}^{\left(q\right)}+\alpha_{2,1}^{\left(p,q\right)}\sum_{j=1}^{n}Y_{j+1}^{\left(p\right)}Y_{j}^{\left(q\right)}+\alpha_{2,2}^{\left(p,q\right)}\sum_{j=1}^{n}Y_{j+1}^{\left(p\right)}Y_{j+1}^{\left(q\right)}\right)\\ = & \sum_{p,q=1}^{d}(\alpha_{1,1}^{\left(p,q\right)}n{\hat{r}}_{n}^{\left(p,q\right)}\left(0\right)+\alpha_{1,2}^{\left(p,q\right)}\left(n{\hat{r}}_{n}^{\left(p,q\right)}\left(1\right)+\underset{★}{\underset{︸}{Y_{n}^{\left(p\right)}Y_{n+1}^{\left(q\right)}}}\right)+\alpha_{2,1}^{\left(p,q\right)}\left(n{\hat{r}}_{n}^{\left(q,p\right)}\left(1\right)+\underset{★}{\underset{︸}{Y_{n+1}^{\left(p\right)}Y_{n}^{\left(q\right)}}}\right)\\ \; & \;\;+\alpha_{2,2}^{\left(p,q\right)}\left(n{\hat{r}}_{n}^{\left(p,q\right)}\left(0\right)+\underset{★}{\underset{︸}{Y_{n+1}^{\left(p\right)}Y_{n+1}^{\left(q\right)}}}-\underset{★}{\underset{︸}{Y_{1}^{\left(p\right)}Y_{1}^{\left(q\right)}}}\right)), \end{array}$$

Note that for each of the terms labeled by ★, the following holds for $d^{*}\in \left(\frac{1}{4},\frac{1}{2}\right)$:

$$\begin{array}{c} n^{-2d^{*}}★\overset{P}{\to }0,\;\left(n\to \infty \right). \end{array}$$

We use this property later on when dealing with the asymptotics of the term in (A17). Finally, we consider the term in (A17) for h≥3 and arrive at:

(A18) $$\begin{array}{cc} \; & \sum_{j=1}^{n}\sum_{p,q=1}^{d}\sum_{i,k=1}^{h}Y_{j+i-1}^{\left(p\right)}Y_{j+k-1}^{\left(q\right)}\alpha_{ik}^{\left(p,q\right)}\\ = & \sum_{p,q=1}^{d}\sum_{i,k=1}^{h}\alpha_{ik}^{\left(p,q\right)}\sum_{j=i}^{n+i-1}Y_{j}^{\left(p\right)}Y_{j+k-i}^{\left(q\right)}\\ = & \sum_{p,q=1}^{d}\sum_{l=0}^{h-1}\sum_{i=1}^{h-l}\alpha_{i,i+l}^{\left(p,q\right)}\sum_{j=i}^{n+i-1}Y_{j}^{\left(p\right)}Y_{j+l}^{\left(q\right)}+\sum_{p,q=1}^{d}\sum_{l=-(h-1)}^{-1}\sum_{i=1-l}^{h}\alpha_{i,i+l}^{\left(p,q\right)}\sum_{j=i}^{n+i-1}Y_{j}^{\left(p\right)}Y_{j+l}^{\left(q\right)}\\ = & \sum_{p,q=1}^{d}\sum_{l=0}^{h-1}\sum_{i=1}^{h-l}\alpha_{i,i+l}^{\left(p,q\right)}\sum_{j=i}^{n+i-1}Y_{j}^{\left(p\right)}Y_{j+l}^{\left(q\right)}\\ \; & \;\;+\sum_{p,q=1}^{d}\sum_{l=1}^{h-1}\sum_{i=1}^{h-l}\alpha_{i+l,i}^{\left(p,q\right)}\sum_{j=i}^{n+i-1}Y_{j+l}^{\left(p\right)}Y_{j}^{\left(q\right)} \end{array}$$

$$\begin{array}{cc} = & \sum_{p,q=1}^{d}\sum_{i=1}^{h}\alpha_{i,i}^{\left(p,q\right)}\sum_{j=i}^{n+i-1}Y_{j}^{\left(p\right)}Y_{j}^{\left(q\right)} \end{array}$$

(A19) $$\begin{array}{cc} \; & \;\;+\sum_{p,q=1}^{d}\sum_{l=1}^{h-1}\sum_{i=1}^{h-l}\left(\alpha_{i,i+l}^{\left(p,q\right)}\sum_{j=i}^{n+i-1}Y_{j}^{\left(p\right)}Y_{j+l}^{\left(q\right)}+\alpha_{i+l,i}^{\left(p,q\right)}\sum_{j=i}^{n+i-1}Y_{j+l}^{\left(p\right)}Y_{j}^{\left(q\right)}\right)\\ = & \sum_{p,q=1}^{d}\left(\alpha_{1,1}^{\left(p,q\right)}\sum_{j=1}^{n}Y_{j}^{\left(p\right)}Y_{j}^{\left(q\right)}+\sum_{i=2}^{h}\alpha_{i,i}^{\left(p,q\right)}\sum_{j=i}^{n+i-1}Y_{j}^{\left(p\right)}Y_{j}^{\left(q\right)}\right)\\ \; & \;\;+\sum_{p,q=1}^{d}\sum_{l=1}^{h-2}(\left(\alpha_{1,1+l}^{\left(p,q\right)}\sum_{j=1}^{n}Y_{j}^{\left(p\right)}Y_{j+l}^{\left(q\right)}+\alpha_{1+l,1}^{\left(p,q\right)}\sum_{j=1}^{n}Y_{j+l}^{\left(p\right)}Y_{j}^{\left(q\right)}\right)\\ \; & \;\;\;\;\;\;+\sum_{i=2}^{h-l}\left(\alpha_{i,i+l}^{\left(p,q\right)}\sum_{j=i}^{n+i-1}Y_{j}^{\left(p\right)}Y_{j+l}^{\left(q\right)}+\alpha_{i+l,i}^{\left(p,q\right)}\sum_{j=i}^{n+i-1}Y_{j+l}^{\left(p\right)}Y_{j}^{\left(q\right)}\right)) \end{array}$$

(A20) $$\begin{array}{cc} \; & \;\;+\sum_{p,q=1}^{d}\left(\alpha_{1,h}^{\left(p,q\right)}\sum_{j=1}^{n}Y_{j}^{\left(p\right)}Y_{j+h-1}^{\left(q\right)}+\alpha_{h,1}^{\left(p,q\right)}Y_{j+h-1}^{\left(p\right)}Y_{j}^{\left(q\right)}\right)\\ = & \sum_{p,q=1}^{d}\left(\alpha_{1,1}^{\left(p,q\right)}n{\hat{r}}_{n}^{\left(p,q\right)}\left(0\right)+\sum_{i=2}^{h}\alpha_{i,i}^{\left(p,q\right)}\left(\underset{★}{\underset{︸}{\sum_{j=n+1}^{n+i-1}Y_{j}^{\left(p\right)}Y_{j}^{\left(q\right)}}}+n{\hat{r}}_{n}^{\left(p,q\right)}\left(0\right)-\underset{★}{\underset{︸}{\sum_{j=1}^{i-1}Y_{j}^{\left(p\right)}Y_{j}^{\left(q\right)}}}\right)\right)\\ \; & +\sum_{p,q=1}^{d}\sum_{l=1}^{h-2}(\left(\alpha_{1,1+l}^{\left(p,q\right)}n{\hat{r}}_{n}^{\left(p,q\right)}\left(l\right)+\alpha_{1+l,1}^{\left(p,q\right)}n{\hat{r}}_{n}^{\left(q,p\right)}\left(l\right)\right)\\ \; & \;\;\;\;\;\;+\sum_{i=2}^{h-l}(\alpha_{i,i+l}^{\left(p,q\right)}\left(\underset{★}{\underset{︸}{\sum_{j=n-l+1}^{n+i-1}Y_{j}^{\left(p\right)}Y_{j+l}^{\left(q\right)}}}+n{\hat{r}}_{n}^{\left(p,q\right)}\left(l\right)-\underset{★}{\underset{︸}{\sum_{j=1}^{i-1}Y_{j}^{\left(p\right)}Y_{j+l}^{\left(q\right)}}}\right)\\ \; & \;\;\;\;\;\;+\alpha_{i+l,i}^{\left(p,q\right)}\left(\underset{★}{\underset{︸}{\sum_{j=n-l+1}^{n+i-1}Y_{j+l}^{\left(p\right)}Y_{j}^{\left(q\right)}}}+n{\hat{r}}_{n}^{\left(q,p\right)}\left(l\right)-\underset{★}{\underset{︸}{\sum_{j=1}^{i-1}Y_{j+l}^{\left(p\right)}Y_{j}^{\left(q\right)}}}\right))) \end{array}$$

(A21) $$\begin{array}{cc} \; & +\sum_{p,q=1}^{d}(\alpha_{1,h}^{\left(p,q\right)}\left(\underset{★}{\underset{︸}{\sum_{j=n-h+2}^{n}Y_{j}^{\left(p\right)}Y_{j+h-1}^{\left(q\right)}}}+n{\hat{r}}_{n}^{\left(p,q\right)}\left(h-1\right)\right) \end{array}$$

(A22) $$\begin{array}{cc} \; & \;\;\;\;\;\;+\alpha_{h,1}^{\left(p,q\right)}\left(\underset{★}{\underset{︸}{\sum_{j=n-h+2}^{n}Y_{j+h-1}^{\left(p\right)}Y_{j}^{\left(q\right)}}}+n{\hat{r}}_{n}^{\left(q,p\right)}\left(h-1\right)\right)). \end{array}$$

Again for each of the terms labeled by ★ it holds for $d^{*}\in \left(\frac{1}{4},\frac{1}{2}\right)$:

$$\begin{array}{c} n^{-2d^{*}}★\overset{P}{\to }0,\;\left(n\to \infty \right), \end{array}$$

since each ★ describes a sum with a finite number (independent of *n*) of summands. Therefore, we continue to express the terms denoted by ★ by $o_{P}\left(n^{2d^{*}}\right)$. With these calculations, we are able to re-express the partial sum, whose asymptotics we are interested in, in terms of the sample cross-correlations of the original long-range dependent process ${\left(Y_{j}\right)}_{j\in Z}$. Finally, the previous calculations lead to:

(A23) $$\begin{array}{cc} \; & \sum_{j=1}^{n}f\left(Y_{j,h}\right)\\ \overset{D}{=}\;\;\; & \sum_{j=1}^{n}f^{*}\left(U_{j,h}\right)\\ \overset{\left(A14\right)}{=} & \sum_{j=1}^{n}\left(\sum_{1\leq i,k\leq dh}E\left(f^{*}\left(U_{j,h}\right)U_{j,h}^{\left(i\right)}U_{j,h}^{\left(k\right)}\right)U_{j,h}^{\left(i\right)}U_{j,h}^{\left(k\right)}-\sum_{i=1}^{dh}E\left(f^{*}\left(U_{j,h}\right){\left(U_{j,h}^{\left(i\right)}\right)}^{2}\right)\right)+o_{P}\left(n^{2d^{*}}\right)\\ \overset{D}{\underset{\left(A17\right)}{=}} & \sum_{j=1}^{n}\sum_{p,q=1}^{d}\sum_{i,k=1}^{h}\alpha_{ik}^{\left(p,q\right)}\left(Y_{j+i-1}^{\left(p\right)}Y_{j+k-1}^{\left(q\right)}-E\left(Y_{j+i-1}^{\left(p\right)}Y_{j+k-1}^{\left(q\right)}\right)\right)+o_{P}\left(n^{2d^{*}}\right), \end{array}$$

where (A23) follows, since (A15) yields:

$$\begin{array}{cc} \sum_{i=1}^{dh}E\left(f^{*}\left(U_{j,h}\right){\left(U_{j,h}^{\left(i\right)}\right)}^{2}\right)=\;\;\; & \sum_{1\leq i,k\leq dh}E\left(f^{*}\left(U_{j,h}\right)U_{j,h}^{\left(i\right)}U_{j,h}^{\left(k\right)}\right)E\left(U_{j,h}^{\left(i\right)}U_{j,h}^{\left(k\right)}\right)\\ \overset{\left(A17\right)}{=} & \sum_{p,q=1}^{d}\sum_{i,k=1}^{h}\alpha_{ik}^{\left(p,q\right)}E\left(Y_{j+i-1}^{\left(p\right)}Y_{j+k-1}^{\left(q\right)}\right). \end{array}$$

Taking the parts containing the sample cross-correlations into account, we derive:

(A24) $$\begin{array}{cc} \; & \sum_{j=1}^{n}\sum_{p,q=1}^{d}\sum_{i,k=1}^{h}\alpha_{ik}^{\left(p,q\right)}\left(Y_{j+i-1}^{\left(p\right)}Y_{j+k-1}^{\left(q\right)}-E\left(Y_{j+i-1}^{\left(p\right)}Y_{j+k-1}^{\left(q\right)}\right)\right)+o_{P}\left(n^{2d^{*}}\right)\\ \overset{\left(A22\right)}{=} & \sum_{p,q=1}^{d}\left(\alpha_{1,1}^{\left(p,q\right)}n\left({\hat{r}}_{n}^{\left(p,q\right)}\left(0\right)-r^{\left(p,q\right)}\left(0\right)\right)+\sum_{i=2}^{h}\alpha_{i,i}^{\left(p,q\right)}n\left({\hat{r}}_{n}^{\left(p,q\right)}\left(0\right)-r^{\left(p,q\right)}\left(0\right)\right)\right)\\ \; & +\sum_{p,q=1}^{d}\sum_{l=1}^{h-2}(\left(\alpha_{1,1+l}^{\left(p,q\right)}n\left({\hat{r}}_{n}^{\left(p,q\right)}\left(l\right)-r^{\left(p,q\right)}\left(l\right)\right)+\alpha_{1+l,1}^{\left(p,q\right)}n\left({\hat{r}}_{n}^{\left(q,p\right)}\left(l\right)-r^{\left(q,p\right)}\left(l\right)\right)\right)\\ \; & \;\;\;\;\;\;+\sum_{i=2}^{h-l}\left(\alpha_{i,i+l}^{\left(p,q\right)}n\left({\hat{r}}_{n}^{\left(p,q\right)}\left(l\right)-r^{\left(p,q\right)}\left(l\right)\right)+\alpha_{i+l,i}^{\left(p,q\right)}n\left({\hat{r}}_{n}^{\left(q,p\right)}\left(l\right)-r^{\left(q,p\right)}\left(l\right)\right)\right))\\ \; & +\sum_{p,q=1}^{d}\left(\alpha_{1,h}^{\left(p,q\right)}n\left({\hat{r}}_{n}^{\left(p,q\right)}\left(h-1\right)-r^{\left(p,q\right)}\left(h-1\right)\right)+\alpha_{h,1}^{\left(p,q\right)}n\left({\hat{r}}_{n}^{\left(q,p\right)}\left(h-1\right)-r^{\left(q,p\right)}\left(h-1\right)\right)\right)\\ \; & +o_{P}\left(n^{2d^{*}}\right) \end{array}$$

(A25) $$\begin{array}{cc} =\;\;\; & n\sum_{p,q=1}^{d}\left(\sum_{l=0}^{h-1}\sum_{i=1}^{h-l}\alpha_{i,i+l}^{\left(p,q\right)}\left({\hat{r}}_{n}^{\left(p,q\right)}\left(l\right)-r^{\left(p,q\right)}\left(l\right)\right)+\sum_{l=1}^{h-1}\sum_{i=1}^{h-l}\alpha_{i+l,i}^{\left(p,q\right)}\left({\hat{r}}_{n}^{\left(q,p\right)}\left(l\right)-r^{\left(q,p\right)}\left(l\right)\right)\right))\\ \; & \;+o_{P}\left(n^{2d^{*}}\right). \end{array}$$

We take a closer look at the impact of each long-range dependence parameter $d_{p}$, p=1,…,d to the convergence of this sum. The setting we are considering does not allow for a normalization depending on *p* and *q* for each cross-correlation $\left({\hat{r}}_{n}^{\left(p,q\right)}\left(l\right)-r^{\left(p,q\right)}\left(l\right)\right)$, l=0,…,h−1, but we need to find a normalization for all p,q=1,…,d. Hence, we need to remember the set $P^{*}:=\left\{p\in \left\{1,\ldots ,d\right\}:\;d_{p}\geq d_{q}\forall q\in \left\{1,\ldots ,d\right\}\right\}$ and the parameter $d^{*}=\max_{p=1,\ldots ,d}d_{p}$, such that for each $p\in P^{*}$, we have $d_{p}=d^{*}$. For each p,q∈{1,…,d} with $\left(p,q\right)\notin \left(P^{*}\times P^{*}\right)$ and l=0,…,h−1, we conclude that:

(A26) $$\begin{array}{cc} E\left({\left(n^{1-2d^{*}}\left({\hat{r}}_{n}^{\left(p,q\right)}\left(l\right)-r^{\left(p,q\right)}\left(l\right)\right)\right)}^{2}\right) & =n^{2\left(d_{p}+d_{q}-2d^{*}\right)}E\left({\left(n^{1-d_{p}-d_{q}}\left({\hat{r}}_{n}^{\left(p,q\right)}\left(l\right)-r^{\left(p,q\right)}\left(l\right)\right)\right)}^{2}\right)\\ \; & =n^{2d_{p}+2d_{q}-4d^{*}}C_{2}\left(L_{p,p}L_{q,q}+L_{p,q}L_{q,p}\right)\\ \; & \overset{\left(n\to \infty \right)}{\to }0, \end{array}$$

since $d_{p}+d_{q}-2d^{*}<0$. This implies that:

$$\begin{array}{c} n^{1-2d^{*}}\left({\hat{r}}_{n}^{\left(p,q\right)}\left(0\right)-r^{\left(p,q\right)}\left(0\right)\right)\overset{P}{\to }0 \end{array}$$

Hence, using Slutsky’s theorem, the crucial parameters that determine the normalization and therefore, the limit distribution of (A27) are given in $P^{*}$. We have an equal long-range dependence parameter $d^{*}$ to regard for all $p\in P^{*}$. Applying Lemma A1, we obtain the following, by using the symmetry in l=0 of the cross-correlation function $r^{\left(p,q\right)}\left(0\right)=r^{\left(q,p\right)}\left(0\right)$ for $p,q\in P^{*}$:

(A27) $$\begin{array}{cc} \; & \sum_{p,q=1}^{d}\left(\sum_{l=0}^{h-1}\sum_{i=1}^{h-l}\alpha_{i,i+l}^{\left(p,q\right)}\left({\hat{r}}_{n}^{\left(p,q\right)}\left(l\right)-r^{\left(p,q\right)}\left(l\right)\right)+\sum_{l=1}^{h-1}\sum_{i=1}^{h-l}\alpha_{i+l,i}^{\left(p,q\right)}\left({\hat{r}}_{n}^{\left(q,p\right)}\left(l\right)-r^{\left(q,p\right)}\left(l\right)\right)\right))\\ = & \sum_{p,q\in P^{*}}\left(\sum_{l=0}^{h-1}\sum_{i=1}^{h-l}\alpha_{i,i+l}^{\left(p,q\right)}\left({\hat{r}}_{n}^{\left(p,q\right)}\left(0\right)-r^{\left(p,q\right)}\left(0\right)\right)+\sum_{l=1}^{h-1}\sum_{i=1}^{h-l}\alpha_{i+l,i}^{\left(p,q\right)}\left({\hat{r}}_{n}^{\left(q,p\right)}\left(0\right)-r^{\left(q,p\right)}\left(0\right)\right)\right))\\ \; & \;+o_{P}\left(n^{2d^{*}-1}\right)\\ = & \sum_{p,q\in P^{*}}\left({\hat{r}}_{n}^{\left(p,q\right)}\left(0\right)-r^{\left(p,q\right)}\left(0\right)\right)\left(\sum_{l=0}^{h-1}\sum_{i=1}^{h-l}\alpha_{i,i+l}^{\left(p,q\right)}+\sum_{l=1}^{h-1}\sum_{i=1}^{h-l}\alpha_{i+l,i}^{\left(p,q\right)}\right)+o_{P}\left(n^{2d^{*}}\right)\\ = & \sum_{p,q\in P^{*}}\left({\hat{r}}_{n}^{\left(p,q\right)}\left(0\right)-r^{\left(p,q\right)}\left(0\right)\right)\left(\sum_{i,k=1}^{h}\alpha_{i,k}^{\left(p,q\right)}\right)+o_{P}\left(n^{2d^{*}}\right)\\ = & \sum_{p,q\in P^{*}}{\tilde{\alpha }}^{\left(p,q\right)}\left({\hat{r}}_{n}^{\left(p,q\right)}\left(0\right)-r^{\left(p,q\right)}\left(0\right)\right)+o_{P}\left(n^{2d^{*}}\right), \end{array}$$

by defining ${\tilde{\alpha }}^{\left(p,q\right)}:=\sum_{i,k=1}^{h}\alpha_{i,k}^{\left(p,q\right)}$. Applying the continuous mapping theorem given in [33], Theorem 2.3, to the result in Corollary A1, we arrive at:

$$\begin{array}{cc} n^{-2d^{*}}{\left(C_{2}\right)}^{-1/2}\sum_{j=1}^{n}f\left(Y_{j,h}\right) & =n^{-2d^{*}}\left(n\sum_{p,q=1}^{d}{\tilde{\alpha }}^{\left(p,q\right)}\left({\hat{r}}_{n}^{\left(p,q\right)}\left(0\right)-r^{\left(p,q\right)}\left(0\right)\right)+o_{P}\left(n^{2d^{*}}\right)\right)\\ \; & =n^{1-2d^{*}}{\left(C_{2}\right)}^{-1/2}\sum_{p,q=1}^{d}{\tilde{\alpha }}^{\left(p,q\right)}\left({\hat{r}}_{n}^{\left(p,q\right)}\left(0\right)-r^{\left(p,q\right)}\left(0\right)\right)+o_{P}\left(1\right)\\ \; & \overset{D}{\to }\sum_{p,q\in P^{*}}{\tilde{\alpha }}^{\left(p,q\right)}Z_{2,d^{*}+1/2}^{\left(p,q\right)}\left(1\right), \end{array}$$

where:

$$\begin{array}{c} Z_{2,d^{*}+1/2}^{\left(p,q\right)}\left(1\right)=K_{p,q}\left(d^{*}\right)\int_{R^{2}}^{\prime \prime }\frac{\exp\left(i\left(\lambda_{1}+\lambda_{2}\right)\right)-1}{i\left(\lambda_{1}+\lambda_{2}\right)}{\left|\lambda_{1}\lambda_{2}\right|}^{-d^{*}}{\tilde{B}}_{L}^{\left(p\right)}\left(d\lambda_{1}\right){\tilde{B}}_{L}^{\left(q\right)}\left(d\lambda_{2}\right). \end{array}$$

The matrix $K\left(d^{*}\right)$ is given in Corollary A1. Moreover, ${\tilde{B}}_{L}\left(d\lambda \right)$ is a multivariate Hermitian–Gaussian random measure with $E\left(B_{L}\left(d\lambda \right)B_{L}{\left(d\lambda \right)}^{*}\right)=L\;d\lambda$ and *L* as defined in (2). □

### Appendix A.3. Proof of Corollary 2.2

**Proof.** We assumed $d^{*}\in \left(\frac{1}{4},\frac{1}{2}\right)$, because otherwise we leave the long-range dependent setting, since we are studying functionals with Hermite rank 2 and the transformed process would no longer be long-range dependent and limit theorems for functionals of short-range dependent processes would hold, as can be seen in Theorem 4 in [14]. This choice of $d^{*}$ assures that the multivariate generalization of the Reduction Theorem as it is used in the proof of Theorem 2.1 still holds for these softened assumptions, as explained in (8). We turn to the asymptotics of $g^{\left(p,q\right)}\left(Y_{j}\right)$. We obtain for all $p,q\in \left\{1,\ldots ,d\right\}\setminus P^{*}$, i.e., excluding $d_{p}=d_{q}=d^{*}$ and for all l=0,…,h−1 as in (A26), that:

(A28) $$\begin{array}{cc} E\left({\left(n^{1-2d^{*}}\left({\hat{r}}_{n}^{\left(p,q\right)}\left(l\right)-r^{\left(p,q\right)}\left(l\right)\right)\right)}^{2}\right) & =n^{2\left(d_{p}+d_{q}-2d^{*}\right)}E\left({\left(n^{1-d_{p}-d_{q}}\left({\hat{r}}_{n}^{\left(p,q\right)}\left(l\right)-r^{\left(p,q\right)}\left(l\right)\right)\right)}^{2}\right)\\ \; & =n^{2d_{p}+2d_{q}-4d^{*}}C_{2}\left(L_{p,p}L_{q,q}+L_{p,q}L_{q,p}\right)\\ \; & \overset{\left(n\to \infty \right)}{\to }0, \end{array}$$

since $d_{p}+d_{q}-2d^{*}<0$. This implies that:

$$\begin{array}{c} n^{1-2d^{*}}\left({\hat{r}}_{n}^{\left(p,q\right)}\left(0\right)-r^{\left(p,q\right)}\left(0\right)\right)\overset{P}{\to }0. \end{array}$$

Applying Slutsky’s theorem, we observe that only $p,q\in P^{*}$ have an impact on the convergence behavior as it is given in (A27) and hence, the result in Theorem 2.1 holds. □

### Appendix A.4. Proof of Theorem 2.3

**Proof.** We follow the proof of Theorem 2.1 until (A27), in order to obtain a limit distribution that can be expressed by the sum of two standard Rosenblatt random variables:

(A29) $$\begin{array}{cc} \; & \sum_{p,q=1}^{2}{\tilde{\alpha }}^{\left(p,q\right)}\left({\hat{r}}_{n}^{\left(p,q\right)}\left(0\right)-r^{\left(p,q\right)}\left(0\right)\right)\\ = & \frac{1}{n}\sum_{j=1}^{n}\sum_{p,q=1}^{2}{\tilde{\alpha }}^{\left(p,q\right)}\left(Y_{j}^{\left(p\right)}Y_{j}^{\left(q\right)}-r^{\left(p,q\right)}\left(0\right)\right)\\ = & \frac{1}{n}\sum_{j=1}^{n}\left(Y_{j}^{\left(1\right)},Y_{j}^{\left(2\right)}\right)\left(\begin{array}{ccc} {\tilde{\alpha }}^{\left(1,1\right)} & \; & {\tilde{\alpha }}^{\left(1,2\right)}\\ {\tilde{\alpha }}^{\left(2,1\right)} & \; & {\tilde{\alpha }}^{\left(2,2\right)} \end{array}\right){\left(Y_{j}^{\left(1\right)},Y_{j}^{\left(2\right)}\right)}^{t}\\ \; & \;\;\;\;\;\;\;\;\;-E\left(\left(Y_{j}^{\left(1\right)},Y_{j}^{\left(2\right)}\right)\left(\begin{array}{ccc} {\tilde{\alpha }}^{\left(1,1\right)} & \; & {\tilde{\alpha }}^{\left(1,2\right)}\\ {\tilde{\alpha }}^{\left(2,1\right)} & \; & {\tilde{\alpha }}^{\left(2,2\right)} \end{array}\right){\left(Y_{j}^{\left(1\right)},Y_{j}^{\left(2\right)}\right)}^{t}\right). \end{array}$$

We remember that ${\tilde{\alpha }}^{\left(p,q\right)}=\sum_{i,k=1}^{h}\alpha_{i,k}^{\left(p,q\right)}=\sum_{i,k=1}^{h}\alpha_{i+(p-1)h,k+(q-1)h}$ for p,q=1,2 and $A={\left(\alpha_{i,k}\right)}_{1\leq i,k\leq 2h}=\Sigma_{2,h}^{-1}C\Sigma_{2,h}^{-1}$. Since $\Sigma_{2,h}^{-1}$ is the inverse of the covariance matrix $\Sigma_{2,h}$ of $Y_{1,h}$ it is a symmetric matrix. The matrix of second order Hermite coefficients *C* has the representation $C=E\left(Y_{j,h}f\left(Y_{j,h}\right)Y_{j,h}^{t}\right)$ and therefore, $c_{i,k}=E\left(Y_{j,h}^{\left(i\right)}Y_{j,h}^{\left(k\right)}f\left(Y_{j,h}\right)\right)=c_{k,i}$ for each i,k=1,…,2h. Then, A is also a symmetric matrix, since $A^{t}={\left(\Sigma_{2,h}^{-1}C\Sigma_{2,h}^{-1}\right)}^{t}={\left(\Sigma_{2,h}^{-1}\right)}^{t}C^{t}{\left(\Sigma_{2,h}^{-1}\right)}^{t}=A$. We can now show that $\left(\begin{array}{ccc} {\tilde{\alpha }}^{\left(1,1\right)} & & {\tilde{\alpha }}^{\left(1,2\right)}\\ {\tilde{\alpha }}^{\left(2,1\right)} & & {\tilde{\alpha }}^{\left(2,2\right)} \end{array}\right)$ is a symmetric matrix, i.e., ${\tilde{\alpha }}^{\left(1,2\right)}={\tilde{\alpha }}^{\left(2,1\right)}$. To this end, we define $I_{p}={\left(0,0,\ldots ,0,1,\ldots ,1,0,\ldots ,0\right)}^{t}\in R^{2h}$ such that $I_{p}^{\left(i\right)}=1$ only if i=(p−1)h+1,…,ph, p=1,2. Then, we obtain:

$$\begin{array}{c} {\tilde{\alpha }}^{\left(1,2\right)}=\sum_{i,k=1}^{h}\alpha_{i,k}^{\left(1,2\right)}={\left({\tilde{\alpha }}^{\left(1,2\right)}\right)}^{t}={\left(I_{1}^{t}AI_{2}\right)}^{t}=I_{2}^{t}AI_{1}={\tilde{\alpha }}^{\left(2,1\right)}. \end{array}$$

We now apply the new assumption that $r^{\left(1,1\right)}\left(l\right)=r^{\left(2,2\right)}\left(l\right)$, for l=0,…,h−1 and show ${\tilde{\alpha }}^{\left(1,1\right)}={\tilde{\alpha }}^{\left(2,2\right)}$ with the symmetry features of the multivariate normal distribution discussed in (2.2) and in (2.3) in [18], since $c_{i,j}=c_{2h-i+1,2h-j+1}$, i,j=1,…,2h. We have to study:

$$\begin{array}{c} {\tilde{\alpha }}^{\left(2,2\right)}={\left(I_{2}^{t}AI_{2}\right)}^{t}=I_{2}^{t}\Sigma_{2,h}^{-1}C\Sigma_{2,h}^{-1}I_{2}. \end{array}$$

Since $\Sigma_{2,h}^{-1}={\left(g_{i,k}\right)}_{1\leq i,k\leq 2h}$ is a symmetric and persymmetric matrix, we have $g_{i,k}=g_{k,i}$ and $g_{i,k}=g_{2h-i+1,2h-k+1}$ for i,k=1,…,2h. Then, we obtain:

$$\begin{array}{cc} I_{2}^{t}\Sigma_{2,h}^{-1} & =\left(\sum_{i=h+1}^{2h}g_{i,1},\ldots ,\sum_{i=h+1}^{2h}g_{i,2h}\right)\\ \; & =\left(\sum_{i=1}^{h}g_{i+h,1},\ldots ,\sum_{i=1}^{h}g_{i+h,2h}\right)\\ \; & =\left(\sum_{i=1}^{h}g_{h-i+1,2h},\ldots ,\sum_{i=1}^{h}g_{h-i+1,1}\right)\\ \; & =\left(\sum_{i=1}^{h}g_{i,2h},\ldots ,\sum_{i=1}^{h}g_{i,1}\right)\\ \; & =\left(\sum_{i=1}^{h}g_{2h,i},\ldots ,\sum_{i=1}^{h}g_{1,i}\right)\\ \; & =:\left({\tilde{g}}_{2h},\ldots ,{\tilde{g}}_{1}\right). \end{array}$$

Note that:

$$\begin{array}{c} \Sigma_{2,h}^{-1}I_{1}={\left(\sum_{i=1}^{h}g_{1,i},\ldots ,\sum_{i=1}^{h}g_{2h,i}\right)}^{t}={\left({\tilde{g}}_{1},\ldots ,{\tilde{g}}_{2h}\right)}^{t}. \end{array}$$

Then, we arrive at:

$$\begin{array}{cc} {\tilde{\alpha }}^{\left(2,2\right)}={\left(I_{2}^{t}AI_{2}\right)}^{t} & =I_{2}^{t}\Sigma_{2,h}^{-1}C\Sigma_{2,h}^{-1}I_{2}\\ \; & =\sum_{i,k=1}^{2h}{\tilde{g}}_{2h-i+1}{\tilde{g}}_{2h-k+1}c_{i,k}\\ \; & =\sum_{i,k=1}^{2h}{\tilde{g}}_{2h-i+1}{\tilde{g}}_{2h-k+1}c_{2h-i+1,2h-k+1}\\ \; & =\sum_{i,k=1}^{2h}{\tilde{g}}_{i}{\tilde{g}}_{k}c_{i,k}\\ \; & =I_{1}^{t}\Sigma_{2,h}^{-1}C\Sigma_{2,h}^{-1}I_{1}\\ \; & ={\tilde{\alpha }}^{\left(1,1\right)}. \end{array}$$

Therefore, we have to deal with a special type of 2×2 matrix, since the original matrix in the formula (A27), namely $\left(\begin{array}{ccc} {\tilde{\alpha }}^{\left(1,1\right)} & & {\tilde{\alpha }}^{\left(1,2\right)}\\ {\tilde{\alpha }}^{\left(2,1\right)} & & {\tilde{\alpha }}^{\left(2,2\right)} \end{array}\right)$, has now reduced to $\left(\begin{array}{ccc} {\tilde{\alpha }}^{\left(1,1\right)} & & {\tilde{\alpha }}^{\left(1,2\right)}\\ {\tilde{\alpha }}^{\left(1,2\right)} & & {\tilde{\alpha }}^{\left(1,1\right)} \end{array}\right)$. Finally, we know that any real-valued symmetric matrix *A* can be decomposed via diagonalization into an orthogonal matrix *V* and a diagonal matrix *D*, where the entries of the latter one are determined via the eigenvalues of *A*, for details, as can be seen in [34], p. 327. We can explicitly give formulas for the entries of these matrices here:

$$\begin{array}{c} V=\left(\begin{array}{ccc} -2^{-1/2} & \; & 2^{-1/2}\\ 2^{-1/2} & \; & 2^{-1/2} \end{array}\right),\;D=\left(\begin{array}{ccc} \lambda_{1}={\tilde{\alpha }}^{\left(1,1\right)}-{\tilde{\alpha }}^{\left(1,2\right)} & \; & 0\\ 0 & \; & \lambda_{2}={\tilde{\alpha }}^{\left(1,1\right)}+{\tilde{\alpha }}^{\left(1,2\right)} \end{array}\right), \end{array}$$

such that:

$$\begin{array}{c} VDV=\left(\begin{array}{ccc} \frac{\lambda_{1}+\lambda_{2}}{2} & \; & \frac{\lambda_{2}-\lambda_{1}}{2}\\ \frac{\lambda_{2}-\lambda_{1}}{2} & \; & \frac{\lambda_{1}+\lambda_{2}}{2} \end{array}\right)=\left(\begin{array}{ccc} {\tilde{\alpha }}^{\left(1,1\right)} & \; & {\tilde{\alpha }}^{\left(1,2\right)}\\ {\tilde{\alpha }}^{\left(1,2\right)} & \; & {\tilde{\alpha }}^{\left(1,1\right)} \end{array}\right). \end{array}$$

Therefore, continuing with (A29), we now have the representation:

(A30) $$\begin{array}{cc} \; & \frac{1}{n}\sum_{j=1}^{n}\left(Y_{j}^{\left(1\right)},Y_{j}^{\left(2\right)}\right)\left(\begin{array}{ccc} {\tilde{\alpha }}^{\left(1,1\right)} & \; & {\tilde{\alpha }}^{\left(1,2\right)}\\ {\tilde{\alpha }}^{\left(2,1\right)} & \; & {\tilde{\alpha }}^{\left(2,2\right)} \end{array}\right){\left(Y_{j}^{\left(1\right)},Y_{j}^{\left(2\right)}\right)}^{t}\\ \; & \;\;\;\;\;\;\;\;\;-E\left(\left(Y_{j}^{\left(1\right)},Y_{j}^{\left(2\right)}\right)\left(\begin{array}{ccc} {\tilde{\alpha }}^{\left(1,1\right)} & \; & {\tilde{\alpha }}^{\left(1,2\right)}\\ {\tilde{\alpha }}^{\left(2,1\right)} & \; & {\tilde{\alpha }}^{\left(2,2\right)} \end{array}\right){\left(Y_{j}^{\left(1\right)},Y_{j}^{\left(2\right)}\right)}^{t}\right)\\ = & \frac{1}{n}\sum_{j=1}^{n}\left(Y_{j}^{\left(1\right)},Y_{j}^{\left(2\right)}\right)VDV{\left(Y_{j}^{\left(1\right)},Y_{j}^{\left(2\right)}\right)}^{t}-E\left(\left(Y_{j}^{\left(1\right)},Y_{j}^{\left(2\right)}\right)VDV{\left(Y_{j}^{\left(1\right)},Y_{j}^{\left(2\right)}\right)}^{t}\right)\\ = & \frac{1}{n}\sum_{j=1}^{n}\frac{{\tilde{\alpha }}^{\left(1,1\right)}-{\tilde{\alpha }}^{\left(1,2\right)}}{2}\left({\left(Y_{j}^{\left(2\right)}-Y_{j}^{\left(1\right)}\right)}^{2}-E{\left(Y_{j}^{\left(2\right)}-Y_{j}^{\left(1\right)}\right)}^{2}\right)\\ \; & \;\;+\frac{1}{n}\sum_{j=1}^{n}\frac{{\tilde{\alpha }}^{\left(1,1\right)}+{\tilde{\alpha }}^{\left(1,2\right)}}{2}\left({\left(Y_{j}^{\left(1\right)}+Y_{j}^{\left(2\right)}\right)}^{2}-E{\left(Y_{j}^{\left(1\right)}+Y_{j}^{\left(2\right)}\right)}^{2}\right)\\ = & \frac{1}{n}\sum_{j=1}^{n}\left({\tilde{\alpha }}^{\left(1,1\right)}-{\tilde{\alpha }}^{\left(1,2\right)}\right)\left(1-r^{\left(1,2\right)}\left(0\right)\right)\left({\left(\frac{Y_{j}^{\left(2\right)}-Y_{j}^{\left(1\right)}}{\sqrt{2-2r^{\left(1,2\right)}\left(0\right)}}\right)}^{2}-1\right)\\ \; & \;\;+\frac{1}{n}\sum_{j=1}^{n}\left({\tilde{\alpha }}^{\left(1,1\right)}+{\tilde{\alpha }}^{\left(1,2\right)}\right)\left(1+r^{\left(1,2\right)}\left(0\right)\right)\left({\left(\frac{Y_{j}^{\left(1\right)}+Y_{j}^{\left(2\right)}}{\sqrt{2+2r^{\left(1,2\right)}\left(0\right)}}\right)}^{2}-1\right)\\ \; & =\frac{1}{n}\left({\tilde{\alpha }}^{\left(1,1\right)}-{\tilde{\alpha }}^{\left(1,2\right)}\right)\left(1-r^{\left(1,2\right)}\left(0\right)\right)\sum_{j=1}^{n}H_{2}\left(Y_{j}^{*}\right)\\ \; & \;\;+\frac{1}{n}\left({\tilde{\alpha }}^{\left(1,1\right)}+{\tilde{\alpha }}^{\left(1,2\right)}\right)\left(1+r^{\left(1,2\right)}\left(0\right)\right)\sum_{j=1}^{n}H_{2}\left(Y_{j}^{**}\right), \end{array}$$

with $Y_{j}^{*}:=\frac{Y_{j}^{\left(2\right)}-Y_{j}^{\left(1\right)}}{\sqrt{2-2r^{\left(1,2\right)}\left(0\right)}}$ and $Y_{j}^{**}:=\frac{Y_{j}^{\left(1\right)}+Y_{j}^{\left(2\right)}}{\sqrt{2+2r^{\left(1,2\right)}\left(0\right)}}$. Now, note that:

$$\begin{array}{c} E\left(Y_{j}^{*}Y_{j}^{**}\right)=E\left(\frac{Y_{j}^{\left(2\right)}-Y_{j}^{\left(1\right)}}{\sqrt{2-2r^{\left(1,2\right)}\left(0\right)}}\frac{Y_{j}^{\left(1\right)}+Y_{j}^{\left(2\right)}}{\sqrt{2+2r^{\left(1,2\right)}\left(0\right)}}\right)=0. \end{array}$$

Therefore, we created a bivariate long-range dependent Gaussian process, since:

$$\begin{array}{c} \left(\begin{array}{ccc} -1 & \; & 1\\ 1 & \; & 1 \end{array}\right){\left(Y_{j}^{\left(1\right)},Y_{j}^{\left(2\right)}\right)}^{t}={\left(Y_{j}^{*},Y_{j}^{**}\right)}^{t}∼N\left(0,I_{2}\right) \end{array}$$

with cross-covariance function:

(A31) $$\begin{array}{cc} r_{*}^{\left(1,2\right)}\left(k\right):=E\left(Y_{j}^{*}Y_{j+k}^{**}\right) & =E\left(\frac{Y_{j}^{\left(2\right)}-Y_{j}^{\left(1\right)}}{\sqrt{2-2r^{\left(1,2\right)}\left(0\right)}}\frac{Y_{j+k}^{\left(1\right)}+Y_{j+k}^{\left(2\right)}}{\sqrt{2+2r^{\left(1,2\right)}\left(0\right)}}\right)\\ \; & =\frac{r^{\left(2,1\right)}\left(k\right)+r^{\left(2,2\right)}\left(k\right)-r^{\left(1,1\right)}\left(k\right)-r^{\left(1,2\right)}\left(k\right)}{2\sqrt{\left(1-r^{\left(1,2\right)}\left(0\right)\right)\left(1+r^{\left(1,2\right)}\left(0\right)\right)}}\\ \; & ≃\frac{L_{2,2}+L_{2,1}-L_{1,2}-L_{1,1}}{2\sqrt{\left(1-r^{\left(1,2\right)}\left(0\right)\right)\left(1+r^{\left(1,2\right)}\left(0\right)\right)}}k^{2d^{*}-1}. \end{array}$$

Note that the covariance functions have the following asymptotic behavior:

$$\begin{array}{cc} r_{*}^{\left(1,1\right)}\left(k\right):=E\left(Y_{j}^{*}Y_{j+k}^{*}\right) & =E\left(\frac{Y_{j}^{\left(2\right)}-Y_{j}^{\left(1\right)}}{\sqrt{2-2r^{\left(1,2\right)}\left(0\right)}}\frac{Y_{j+k}^{\left(2\right)}-Y_{j+k}^{\left(1\right)}}{\sqrt{2-2r^{\left(1,2\right)}\left(0\right)}}\right)\\ \; & =\frac{r^{\left(2,2\right)}\left(k\right)-r^{\left(2,1\right)}\left(k\right)-r^{\left(1,2\right)}\left(k\right)+r^{\left(1,1\right)}\left(k\right)}{2-2r^{\left(1,2\right)}\left(0\right)}\\ \; & ≃\underset{=:L_{1,1}^{*}}{\underset{︸}{\frac{L_{2,2}-L_{2,1}-L_{1,2}+L_{1,1}}{2-2r^{\left(1,2\right)}\left(0\right)}}}k^{2d^{*}-1} \end{array}$$

and analogously:

$$\begin{array}{c} r_{*}^{\left(2,2\right)}\left(k\right):=E\left(Y_{j}^{**}Y_{j+k}^{**}\right)≃\underset{=:L_{2,2}^{*}}{\underset{︸}{\frac{L_{2,2}+L_{2,1}+L_{1,2}+L_{1,1}}{2+2r^{\left(1,2\right)}\left(0\right)}}}k^{2d^{*}-1}. \end{array}$$

We can now apply the result of [14], Theorem 6, since we created a bivariate Gaussian process with independent entries for fixed *j*. Note that for the function we apply here, namely $\tilde{f}\left(Y_{j}^{*},Y_{j}^{**}\right)=H_{2}\left(Y_{j}^{*}\right)+H_{2}\left(Y_{j}^{**}\right)$ the weighting factors in [14], Theorem 6, reduce to $e_{1,1}=e_{2,2}=1$ and $e_{1,2}=e_{2,1}=0$. These weighting factors fit into the result in [14], (3.6) and (3.7), which even yields the joint convergence of the vector of both univariate summands, $\left(H_{2}\left(Y_{j}^{*}\right),H_{2}\left(Y_{j}^{**}\right)\right)$, suitably normalized to a vector of two (dependent) Rosenblatt random variables. Since the long-range dependence property in [13], Definition 2.1 is more specific than in [14], p. 2259, (3.1) (see the considerations in (8)), we are able to scale the variances of each Rosenblatt random variable to 1 and give the covariance between them, by using the normalization given in [15], Theorem 4.3. We obtain:

$$\begin{array}{cc} \; & n^{-2d*}{\left(2C_{2}\right)}^{-1/2}\left({\tilde{\alpha }}^{\left(1,1\right)}-{\tilde{\alpha }}^{\left(1,2\right)}\right)\left(1-r^{\left(1,2\right)}\left(0\right)\right)\sum_{j=1}^{n}H_{2}\left(Y_{j}^{*}\right)\\ \; & \;\;+n^{-2d*}{\left(2C_{2}\right)}^{-1/2}\left({\tilde{\alpha }}^{\left(1,1\right)}+{\tilde{\alpha }}^{\left(1,2\right)}\right)\left(1+r^{\left(1,2\right)}\left(0\right)\right)\sum_{j=1}^{n}H_{2}\left(Y_{j}^{**}\right)\\ \; & \overset{D}{\to }\left({\tilde{\alpha }}^{\left(1,1\right)}-{\tilde{\alpha }}^{\left(1,2\right)}\right)\left(1-r^{\left(1,2\right)}\left(0\right)\right)L_{1,1}^{*}Z_{2,d^{*}+1/2}^{*}\left(1\right)\\ \; & \;+\left({\tilde{\alpha }}^{\left(1,1\right)}+{\tilde{\alpha }}^{\left(1,2\right)}\right)\left(1+r^{\left(1,2\right)}\left(0\right)\right)L_{2,2}^{*}Z_{2,d^{*}+1/2}^{**}\\ \; & =\left({\tilde{\alpha }}^{\left(1,1\right)}-{\tilde{\alpha }}^{\left(1,2\right)}\right)\frac{L_{2,2}-L_{2,1}-L_{1,2}+L_{1,1}}{2}Z_{2,d^{*}+1/2}^{*}\left(1\right)\\ \; & \;\;+\left({\tilde{\alpha }}^{\left(1,1\right)}+{\tilde{\alpha }}^{\left(1,2\right)}\right)\frac{L_{2,2}+L_{2,1}+L_{1,2}+L_{1,1}}{2}Z_{2,d^{*}+1/2}^{**}\left(1\right) \end{array}$$

with $C_{2}:=\frac{1}{2d^{*}\left(4d^{*}-1\right)}$ being the same normalizing factor as in Theorem 2.1. We observe that $Z_{2,d^{*}+1/2}^{*}\left(1\right)$ and $Z_{2,d^{*}+1/2}^{**}\left(1\right)$ are both standard Rosenblatt random variables. Following Corollary A1, their covariance is given by

$$\begin{array}{cc} \mathrm{Cov}\left(Z_{2,d^{*}+1/2}^{*}\left(1\right),Z_{2,d^{*}+1/2}^{**}\left(1\right)\right) & =\frac{{\left(L_{1,2}^{*}+L_{2,1}^{*}\right)}^{2}}{L_{1,1}^{*}L_{2,2}^{*}}\\ \; & =\frac{2\left({\left(L_{2,2}-L_{1,1}\right)}^{2}\right)}{4\left(1-r^{\left(1,2\right)}\left(0\right)\right)\left(1+r^{\left(1,2\right)}\left(0\right)\right)}{\left(L_{1,1}^{*}L_{2,2}^{*}\right)}^{-1}\\ \; & =\frac{{\left(L_{2,2}-L_{1,1}\right)}^{2}}{{\left(L_{1,1}+L_{2,2}\right)}^{2}-{\left(L_{1,2}+L_{2,1}\right)}^{2}}. \end{array}$$

Note that ${\left(L_{1,1}+L_{2,2}\right)}^{2}-{\left(L_{1,2}+L_{2,1}\right)}^{2}\neq 0$ is fulfilled since $L_{1,1}+L_{2,2}\neq L_{1,2}+L_{2,1}$. □
